# Two Isoforms of *serpent* Containing Either One or Two GATA Zinc Fingers Provide Functional Diversity During *Drosophila* Development

**DOI:** 10.3389/fcell.2021.795680

**Published:** 2022-02-01

**Authors:** Douaa Moussalem, Benoit Augé, Luisa Di Stefano, Dani Osman, Vanessa Gobert, Marc Haenlin

**Affiliations:** ^1^ Molecular, Cellular and Developmental Biology Department (MCD), Center for Integrative Biology (CBI), University of Toulouse, CNRS, UPS, Toulouse, France; ^2^ Faculty of Sciences III, Lebanese University, Tripoli, Lebanon; ^3^ Azm Center for Research in Biotechnology and Its Applications, LBA3B, EDST, Lebanese University, Tripoli, Lebanon

**Keywords:** GATA, Friend of GATA, *Drosophila*, zinc finger, alternative splicing

## Abstract

GATA transcription factors play crucial roles in various developmental processes in organisms ranging from flies to humans. In mammals, GATA factors are characterized by the presence of two highly conserved domains, the N-terminal (N-ZnF) and the C-terminal (C-ZnF) zinc fingers. The *Drosophila* GATA factor Serpent (Srp) is produced in different isoforms that contains either both N-ZnF and C-ZnF (SrpNC) or only the C-ZnF (SrpC). Here, we investigated the functional roles ensured by each of these isoforms during *Drosophila* development. Using the CRISPR/Cas9 technique, we generated new mutant fly lines deleted for one (*ΔsrpNC*) or the other (*ΔsrpC*) encoded isoform, and a third one with a single point mutation in the N-ZnF that alters its interaction with its cofactor, the *Drosophila* FOG homolog U-shaped (Ush). Analysis of these mutants revealed that the Srp zinc fingers are differentially required for Srp to fulfill its functions. While SrpC is essential for embryo to adult viability, SrpNC, which is the closest conserved isoform to that of vertebrates, is not. However, to ensure its specific functions in larval hematopoiesis and fertility, Srp requires the presence of both N- and C-ZnF (SrpNC) and interaction with its cofactor Ush. Our results also reveal that *in vivo* the presence of N-ZnF restricts rather than extends the ability of GATA factors to regulate the repertoire of C-ZnF bound target genes.

## Introduction

GATA factors are DNA binding proteins that were named after the consensus nucleic acid sequence they recognize. They are highly conserved proteins that are present in most eukaryotes, ranging from invertebrates to vertebrates (Lowry and Atchley, 2000). Metazoan GATA genes evolved from two ancestral genes, GATA123 and GATA456 (Gillis et al., 2008; Gillis et al., 2009), and expanded either by two genome duplications in vertebrates, which have six paralogs, or by a specific duplication of GATA456 paralogs, as in the fruit fly *Drosophila melanogaster* that has five GATA genes (Gillis et al., 2008; Gillis et al., 2009).

They play essential roles in many developmental processes by regulating cell proliferation, cell-fate specification and differentiation. In mammals, they ensure critical roles in formation of the ectodermal-derived nervous system, endodermal gastrointestinal tract and liver, as well as mesodermal-derived hematopoietic system, cardiovascular system, gonads, and kidneys (Lentjes et al., 2016; Dobrzycki et al., 2020). Their importance throughout development was further substantiated by genome, exome and transcriptome sequencing that has led to the identification of a huge number of GATA mutations in patients with different biological disorders (Fujiwara and Fujiwara, 2017; Tremblay et al., 2018). The type of disease depends on the affected GATA gene and its expression pattern. For example, GATA1, GATA2 and GATA3 proteins are expressed in hematopoietic cell lineages, and mutations affecting these factors are related to numerous hematological disorders like myelodysplastic syndromes, Emberger syndrome, *ß*-thalassemia and various leukemia (Crispino and Horwitz, 2017). As GATA3 is also expressed in developing and differentiated mammary glands, as well as in embryonic kidney, inner ear and parathyroid glands, its mutations are found in breast cancer (Kouros-Mehr et al., 2008) and in hypoparathyroidism, deafness, and renal dysplasia (HDR) syndrome (Esch et al., 2000). Likewise, mutations affecting the GATA4, GATA5 and GATA6 factors that are expressed during the mammalian heart development, are associated to cardiac diseases (Whitcomb et al., 2020).

Besides a high sequence conservation, mammalian and *Drosophila* GATA factors display functional similarities, as they are implicated in the regulation of similar developmental processes, such as hematopoietic precursor proliferation and maintenance (Patient and McGhee, 2002), blood cell differentiation (Rehorn et al., 1996; Takahashi et al., 1997), cardiomyocyte differentiation (Klinedinst and Bodmer, 2003; Zhao et al., 2008), gut formation and maintenance (Reuter, 1994; Walker et al., 2014; Okumura et al., 2016), fertility (Kyrnlahti et al., 2011; Lepesant et al., 2019) and mammalian liver/*Drosophila* fat body development (Rehorn et al., 1996; Zhao et al., 2005; Watt et al., 2007).

At the structural level, the GATA factors zinc finger domains have a Cys-X2-Cys-X17-Cys-X2-Cys consensus sequence followed by a conserved basic amino acid-containing region necessary for DNA binding. In vertebrates, all the six GATA factors (GATA1 to GATA6) contain two zinc finger domains, referred to as N-ZnF and C-ZnF. DNA-binding is mainly established by the C-ZnF and its adjacent basic C-terminal region (Yang and Evans, 1992; Omichinski et al., 1993). Although dispensable for binding to the GATA-containing DNA motif, the N-ZnF contributes to stabilizing protein/DNA interaction, predominantly on palindromic GATA sequences (Yang and Evans, 1992; Trainor et al., 1996). In addition, it was shown that N-ZnF of GATA2 and GATA3 proteins can bind GATC-containing DNA motif, in a manner that depends on its adjacent basic region (Pedone et al., 1997). Finally, the GATA1 N-ZnF and C-ZnF domains participate in GATA factor’s interactions with other transcriptional regulators (Lowry and Mackay, 2006; DeVilbiss et al., 2016).

All six mammalian GATA factors have in common the presence of these two zinc finger domains that are strongly conserved across evolution. In *Drosophila*, only three of the five GATA factors, Pannier, Serpent and Grain, display these canonical two zinc finger domains (Ramain et al., 1993; Lin et al., 1995; Waltzer et al., 2002) and their amino acid sequences are almost identical to those of the mammalian GATA factors. The two remaining *Drosophila* GATA factors, dGATAd and dGATAe, lack the N-ZnF, and are mainly found in invertebrates (Lowry and Atchley, 2000; Gillis et al., 2009). Importantly, in the N-ZnF, a valine residue required for the interaction between GATA proteins and their cofactors of the Friend of GATA (FOG) family (Crispino et al., 1999), is also essential for the functional interaction of the *Drosophila* GATA factor Serpent (Srp) with the *Drosophila* FOG factor U-shaped (Ush) (Fossett et al., 2003).

Although numerous studies have been carried out to determine the functions played by GATA factors, the contribution of the zinc finger domains during establishment of these functions has been largely overlooked. Conservation of developmental processes between mammals and fly, as well as the structural and functional conservation of GATA factors across evolution, led us to consider the fly as an ideal organism model in which to study the contributions of the zinc finger domains to GATA functions. Among the *Drosophila* GATA factors, Srp provides a unique paradigm to decipher *in vivo* the roles of the GATA zinc finger domains, since Srp proteins are produced by alternative splicing as two different isoforms, containing either the two zinc finger domains (SrpNC), like the vertebrate GATA factors, or only the C-ZnF domain (SrpC) (Waltzer et al., 2002).

In this study, we generate and analyze mutant fly lines devoid of either the SrpNC or SrpC isoform to investigate the functions ensured by each isoform during *Drosophila* development. We found that both isoforms regulate redundantly the gut developmental program and part of embryonic hematopoiesis. We also show that the mammalian-like isoform SrpNC is dispensable for most Srp-dependent developmental processes, although it is specifically required for the maintenance of larval blood cell homeostasis and for female fertility. In addition, we show that all SrpNC specific functions depend on its interaction with its FOG cofactor U-shaped. We find that the SrpC isoform is specifically required for embryonic fat body formation and viability during development, indicating that it regulates different developmental programs than those controlled by SrpNC. Altogether, our results reveal a high degree of functional flexibility played by the GATA zinc fingers to fulfil their various roles throughout development. Also, this work illustrates that, like genome duplication in vertebrates, alternative splicing provides an efficient strategy to generate GATA functional diversity.

## Results

### Splicing of *srp* Generates an Alternate Exon That Is Poorly Conserved

**FIGURE 1:**
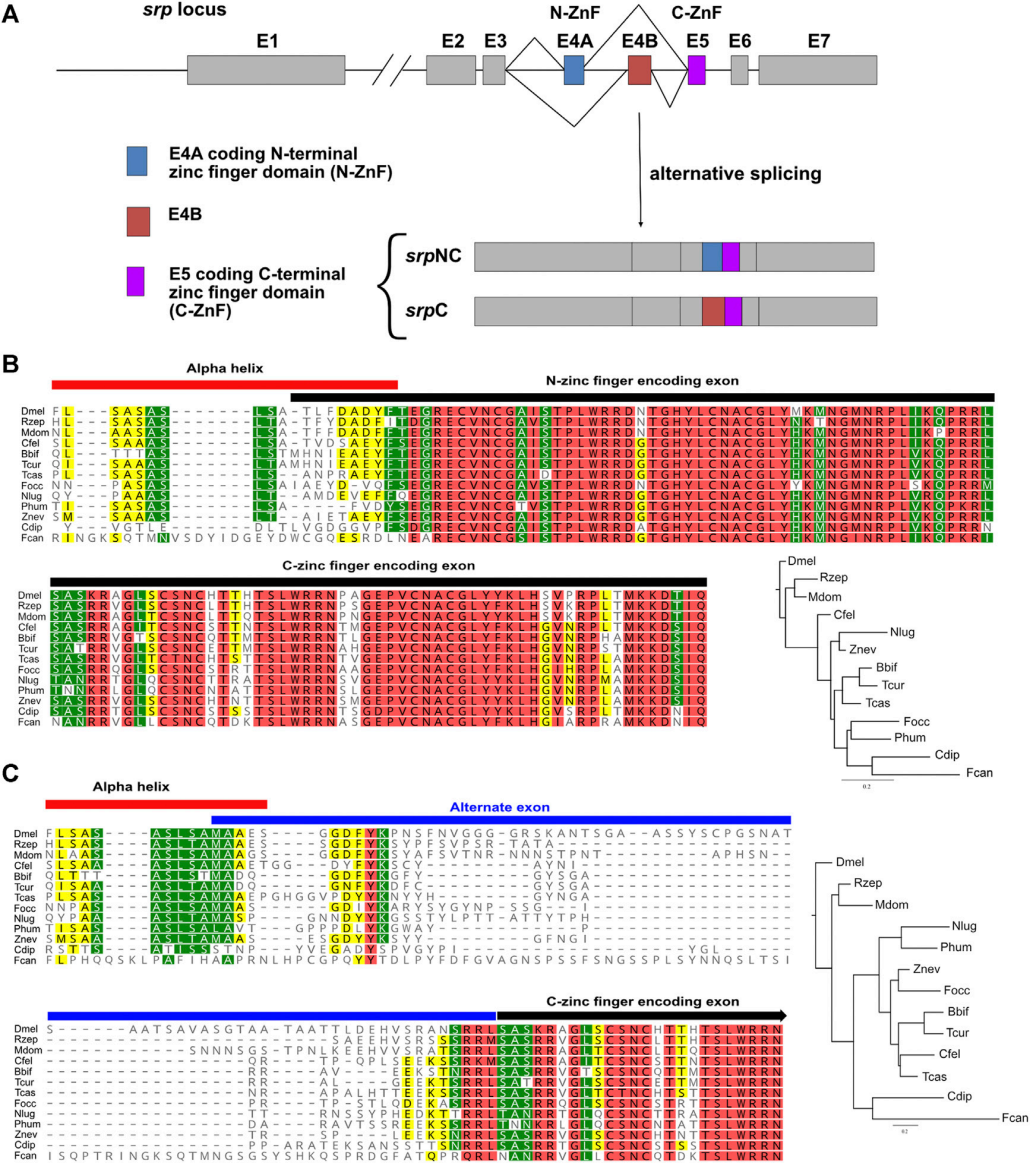
The two Serpent isoforms are conserved in various arthropods. **(A)** Schematic representation of the wild-type *Drosophila melanogaster srp* locus. Exons (E) are represented by boxes and introns by lines. Alternatively spliced exons 4A (E4A) and 4B (E4B) are colored blue and red, respectively, constitutive exon 5 (E5) is colored purple, and all other exons are shown in grey. E4A and E5 code respectively for N-ZnF and C-ZnF domains. Transcripts containing E4A and E5, and those containing E4B and E5 are called *srpNC* and *srpC*, respectively. Alignment of the protein portion of *Drosophila melanogaster* (Dmel) SrpNC encoded by part of exon 3, the N-ZnF encoding exon 4A, and the C-zinc finger encoding exon 5 **(B)**, as well as the portion of Dmel SrpC encoded by part of exon 3, the alternate exon 4B, and the C-ZnF encoding exon 5 **(C)** with sequences from insect orders Diptera *Rhagoletis zephyria* (Rzep) and *Musca domestica* (Mdom), Siphonaptera *Ctenocephalides felis* (Cfel), Hymenoptera *Bombus bifarius* (Bbif) and *Temnothorax curvispinosus* (Tcur), Coleoptera *Tribolium castaneaum* (Tcas), Thysanoptera *Frankliniella occidentalis* (Focc), Hemiptera *Nilaparvata lugens* (Nlug), Psocodea *Pediculus humanus corporis* (Phum), Dyctyoptera *Zootermopsis nevadensis* (Znev), Palaeoptera *Cloeon dipterum* (Cdip) and from the non-insect Hexapod class Collembola *Folsomia candida* (Fcan). Phylogenetic trees are built using FastTree (Geneious prime) from the corresponding alignment. The trees are rooted using the Dmel sequence as the outgroup. Conserved residues are colored according to their similarity: red 100%, green 80–100%, yellow 60–80% and no color less than 60% similar.

Metazoan GATA factors are thought to have evolved from a two-fingered common ancestor (Gillis et al., 2009; Eurmsirilerd and Maduro, 2020). We identified different Srp isoforms, containing either two zinc finger domains (SrpNC) or only one zinc finger domain (SrpC) (Waltzer et al., 2002). Both SrpNC and SrpC contain the C-ZnF, encoded by the fifth exon of *srp*, while *srpNC* transcript results from the inclusion of exon 4A that encodes the Srp N-ZnF, and the simultaneous exclusion of the alternative exon 4B (Figure 1A). On the contrary, *srpC* transcript is obtained by the substitution of the N-ZnF coding exon 4A by the alternative exon 4B that encodes a protein region lacking any known motif. This alternative splicing mechanism leading to the production of the SrpC protein isoform is thought to have arisen in a second evolutionary step, through a specific exon duplication subjected to mutually exclusive splicing (Yue et al., 2016). To trace this event, we performed a phylogenetic analysis of the duplicated exons of *srp* in arthropods. The multiple protein sequence alignment shown in Figure 1B reveals a strong conservation of the two exons encoding the N- and the C- ZnFs between species belonging to various branches of the insect class, in contrast to the alternate exon (E4B in *Drosophila*) included in the *srpC* transcript, which display little similarity with other species (Figure 1C). Apart from the few amino acids located at both ends, which seems to allow the conservation of structural motifs, as well as four to five amino acids located near the beginning of the exon, there is no obvious conservation found within the alternate exon between these species, suggesting that this portion of SrpC might not have any important function.

This observation then raises the question of what are the respective roles of each isoform in the different functions performed by Srp.

### Engineering of *srp* Isoform Specific Loss-Of-Function Mutants

**FIGURE 2:**
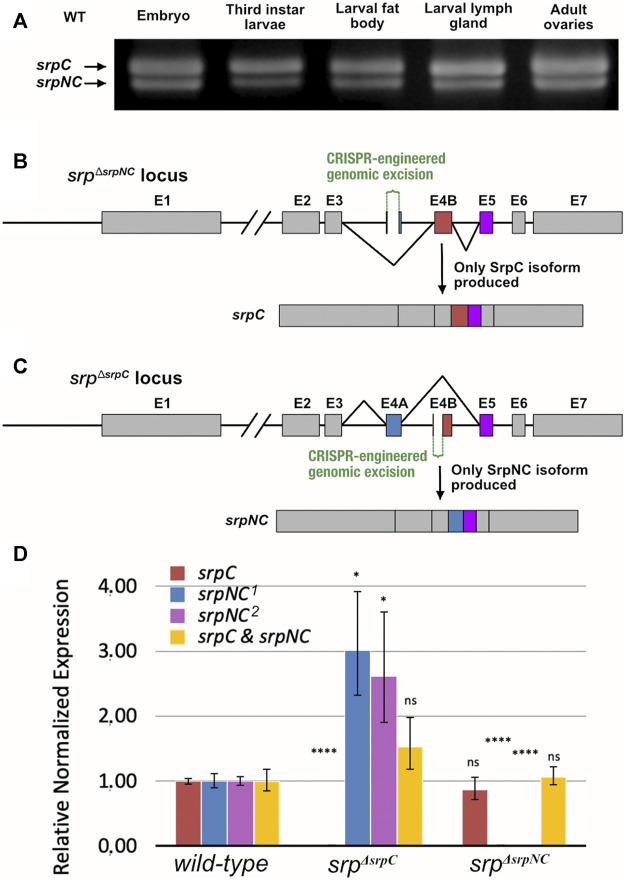
Schematic representation of the *srp* locus and corresponding mutants. **(A)** RT-PCR analysis of wild-type (WT), *srpC* and *srpNC* transcripts from different stages and tissues: ≥ stage12 embryos, whole middle third instar larvae, fat body, lymph gland and young adult female ovaries (the position of the primers is shown in Supplementary Figure S1). Schematic representation of the CRISPR/Cas9-engineered genomic excision corresponding to *srp* ^*ΔsrpNC*^ **(B)** and *srp* ^*ΔsrpC*^ **(C)** mutants. In *srp* ^*ΔsrpNC*^ *,* Exon 4A (E4A), which encodes the N-terminal zinc finger domain, is deleted and only Exon 4B (E4B, red box) is included in the *srp* transcript, and therefore only *srpC* is produced **(B)**. In *srp* ^*ΔsrpC*^, a large part of exon E4B including its splice acceptor site is deleted, so that only splicing including E4A (blue box) can occur, and consequently only the *srpNC* transcript is produced **(C)**. **(D)** qRT-PCR analysis of *srpC, srpNC* and both *srpC+srpNC* mRNAs, in wild-type, *srp* ^*ΔsrpC*^ and *srp* ^*ΔsrpNC*^ embryos at ≥ stage12. The two *srpC* and *srpNC* transcripts are specifically detected by amplification of the exon3-exon4 junction (*srpC* and *srpNC* ^*2*^) or exon 4-exon5 (*srpNC* ^*1*^) in wild-type embryos (reference samples = 1.00), while each of them is lost in the corresponding *srp* ^*ΔsrpC*^ or *srp* ^*ΔsrpNC*^ mutant embryos (the position of the primers is shown in Supplementary Figure S1). Note that *srpNC*, which is less expressed than *srpC* in wild-type embryos (Waltzer et al., 2002; Yue et al., 2016), is about three times more expressed in the *srp* ^*ΔsrpC*^ mutant (≥3.00) compared to wild-type embryos*. p*-values are determined by comparison with the corresponding transcripts from wild-type embryos, ****p ≤0.0001; *p ≤0.05, ns: not significantly different from reference sample (*srp* ^*ΔsrpC*^ embryos: *srpC P* = 3 × 10^−4^ *, srpNC* ^*1*^ *p* = 1.8 × 10^−2^, *srpNC* ^*2*^ *p* = 4.3 × 10^−2^ and both *srpC+srpNC p* = 2.4 × 10^−1^; *srp* ^*ΔsrpNC*^ embryos: *srpC p* = 5.4 × 10^−1^ *, srpNC* ^*1*^ *P* = 1 × 10^−4^, *srpNC* ^*2*^ *p* = 3.6 × 10^−5^ and both *srpC+srpNC p* = 7.7 × 10^−1^), and error bars correspond to standard error of the mean (SEM).

In previous work, we showed that the mRNA isoform *srpC* is at least three times more expressed than *srpNC* throughout embryogenesis (Waltzer et al., 2002). As shown in Figure 2A, both mRNA *srp* isoforms are expressed in all third-instar larvae, including organs already known to express high Srp levels, such as fat body and lymph gland, as well as in adult ovaries (Jung et al., 2005; Senger et al., 2006; Lepesant et al., 2019). Altogether, our data indicate that in different tissues and at different developmental stages, the alternative splicing mechanism occurs, generating two products encoding either one or two zinc finger domains. Thus, their simultaneous presence provides a unique opportunity to compare *in vivo* the role of these two isoforms and hence the specific contributions of each GATA zinc finger domain to Srp functions. Therefore, we generated new mutant fly lines that carry loss-of-function of either *srpNC* or *srpC* using the CRISPR/Cas9 system. It is known that FOG cofactors regulate GATA transcription factors by interacting specifically with the N-ZnF (Fox et al., 1999). It has been shown that the valine residue present in the N-Znf is required for interaction with FOG, and substitution of this valine to glycine alters the association GATA/FOG (Crispino et al., 1999). In *Drosophila*, replacement of the corresponding valine to glycine in the N-ZnF of Srp also alters its functional interaction with the *Drosophila* FOG cofactor U-shaped (Ush) and prevents the function of the Srp/Ush complex as shown by (Fossett et al., 2003). As Ush has been also shown to be involved in several aspects of hematopoiesis and particularly in lamellocyte differentiation (Sorrentino et al., 2007; Avet-Rochex et al., 2010; Banerjee et al., 2019) we also generated a fly line called *srp* ^*V735G*^ harboring the valine to glycine substitution.

We produced mutant flies containing either a deletion of most of exon 4A, preventing the production of the *srpNC* transcript, named *srp* ^*ΔsrpNC*^, or a deletion removing the region containing the splice acceptor site of exon 4B, which prevents production of the *srpC* transcript, named *srp* ^*ΔsrpC*^ (Figures 2B,C respectively). To validate *srpNC* or *srpC* loss in these lines, total RNA was extracted from homozygous embryos for each genotype, and quantitative RT-PCR (qRT-PCR) was performed with primers specific for either *srpC*, *srpNC* or both together (Figure 2D, Supplementary Figure S1). Analysis of these qRT-PCR products confirmed the specific loss of *srpC* or *srpNC* expression in *srp* ^*ΔsrpC*^ or *srp* ^*ΔsrpNC*^ mutant embryos, respectively (Figure 2D, Supplementary Figure S1). Of note, the overall transcription level is not significantly affected in *srp* ^*ΔsrpC*^ mutant (Figure 2D), even though deprivation of *srpC* transcript in *srp* ^*ΔsrpC*^ mutant embryos leads to an approximately threefold increase in the expression of the *srpNC* transcript, compared to controlembryos (Figure 2D). This data confirms that the transcription level at the *srp* locus is not affected in *srp* mutant backgrounds and that the splicing occurs independently of *srp* transcription, as previously published (Yue et al., 2016).

### SrpC, but Not SrpNC, Is Required for Fly Viability

**FIGURE 3:**
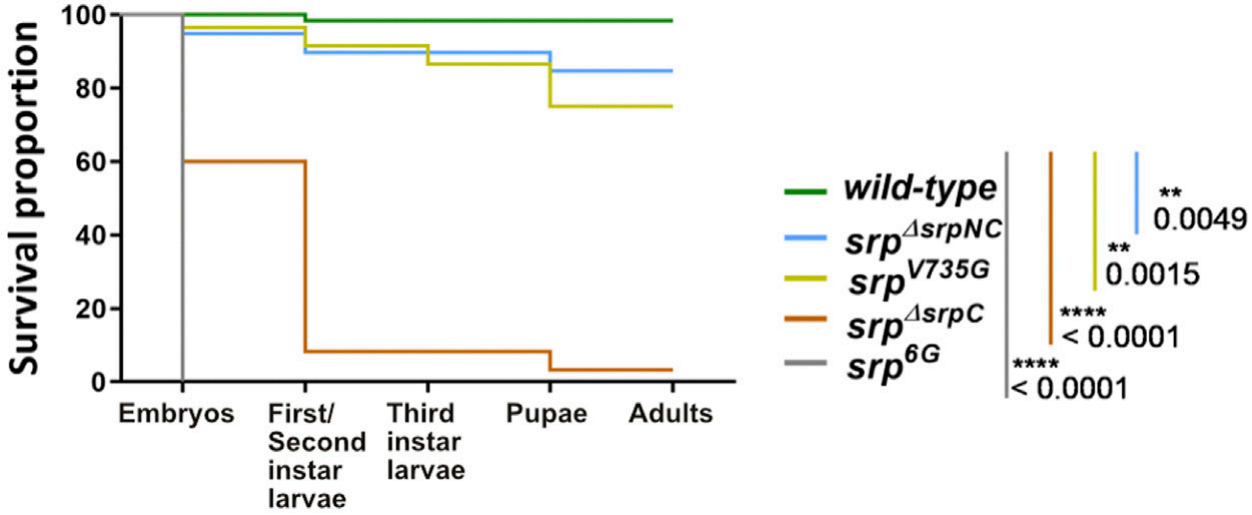
SrpC, but not SrpNC, is required for *Drosophila* development. Kaplan-Meier survival curves of wild-type (green), *srp* ^*ΔsrpNC*^ (blue, *srpNC* depletion), *srp* ^*V735G*^ (yellow, amino-acid substitution in the N-ZnF domain of *srp* protein), *srp* ^*ΔsrpC*^ (brown, *srpC* transcript depletion) and *srp* ^*6G*^ (grey, loss-of-function of both isoforms) embryos. Loss of *srpC* transcript but not *srpNC* mRNA nor expression of V735G-*srpNC* mutated version strongly impacts fly development. *p*-values represent results of a Gehan-Breslon-Wilcoxon test comparing the wild-type with each of the different conditions, indicated by their specific colors (*n* = 60 for each condition).

Loss of *srp* function affects the ability of the fly to reach adulthood, and all embryos homozygous for the null allele *srp* ^*6G*^ die before hatching (Rehorn et al., 1996). To determine whether this lethality is due to the absence of SrpC, SrpNC products, or their simultaneous loss, the viability of *srp* ^*ΔsrpNC*^ and *srp* ^*ΔsrpC*^ mutant embryos was assessed. Interestingly, loss of SrpNC function (*srp* ^*ΔsrpNC*^) is dispensable for flies to develop until the adult stage (83.3% of laid embryos develop until adulthood), while most of *srp* ^*ΔsrpC*^ homozygous embryos die during embryonic and early larval stages (Figure 3, Supplementary Figure S2). Only 8.3% of *srp* ^*ΔsrpC*^ mutant embryos were able to develop until the pupal stage, and the very few escapers that emerged as adults died immediately (Figure 3, Supplementary Figure S2). Moreover, specific downregulation of *srpC* by RNAi during embryogenesis using a ubiquitous Gal4 driver induces a drop in viability like that observed for *srp* ^*ΔsrpC*^ mutants (Supplementary Figure S3C). These results indicate that the SrpC isoform is required for fly viability at all stages of development.

To assess whether presence of only one functional copy of the isoforms can impair viability, the *srp* ^*ΔsrpC*^ and *srp* ^*ΔsrpNC*^ mutant alleles were crossed to flies carrying the null allele *srp* ^*6G*^. 40% of the homozygous *srp* ^*ΔsrpC*^ mutant embryos, which contain two *srpNC* functional copies, die before reaching the first instar larval stage, and removing one *srpNC* functional copy as in *srp* ^*ΔsrpC*^/*srp* ^*6G*^ embryos, results in a further drop in viability with only 5% viable first instar larvae (Figure 3, Supplementary Figure S2). Instead, the presence of only one functional copy of *srpC* can still ensure the viability of most larvae as 85% of *srp* ^*ΔsrpNC*^/*srp* ^*6G*^ embryos reach the first instar larval stage, and most of them develop until adulthood (Supplementary Figure S2).

Similarly, most of the homozygous *srp* ^*V735G*^ (75%) or the *srp* ^*V735G*^/*srp* ^*6G*^ (80%) mutant embryos further develop until adulthood (Figure 3, Supplementary Figure S2). Thus, it appears that even though *srpNC* mRNA is about three time more expressed than in the wild-type, in the *srp* ^*ΔsrpC*^ background the corresponding SrpNC protein isoform is unable to compensate the loss of SrpC product, indicating that the single zinc-finger variant SrpC ensures distinct and essential functions. Hence, the two isoforms are not fully redundant and can play either specific or common roles. We analyzed also mutant combinations of *srp* ^*ΔsrpC*^ with *srp* ^*3*^, an allele that carries a missense mutation in the *srp* exon encoding the C-terminal zinc finger domain, which prevents its interaction with DNA (Rehorn et al., 1996). All embryos of the mutant genotype *srp* ^*ΔsrpC*^ */srp* ^*3*^ die at embryonic stage (Supplementary Figure S2), although a wild-type gene copy of *srpNC* is present in this background, suggesting that the product of *srp* ^*3*^ may antagonize the activities of the SrpNC protein by sequestering it. Furthermore, this result also shows that some genes required for viability are specific targets of SrpC, supporting the hypothesis that for each Srp protein isoform there is also a distinct repertoire of target genes. To further investigate this hypothesis, we explored the contribution of each isoform to the different developmental processes known to be controlled by *srp*.

### SrpC and SrpNC Isoforms Have Redundant Function for Embryonic Gut Development but Not for Fat Body Formation

**FIGURE 4:**
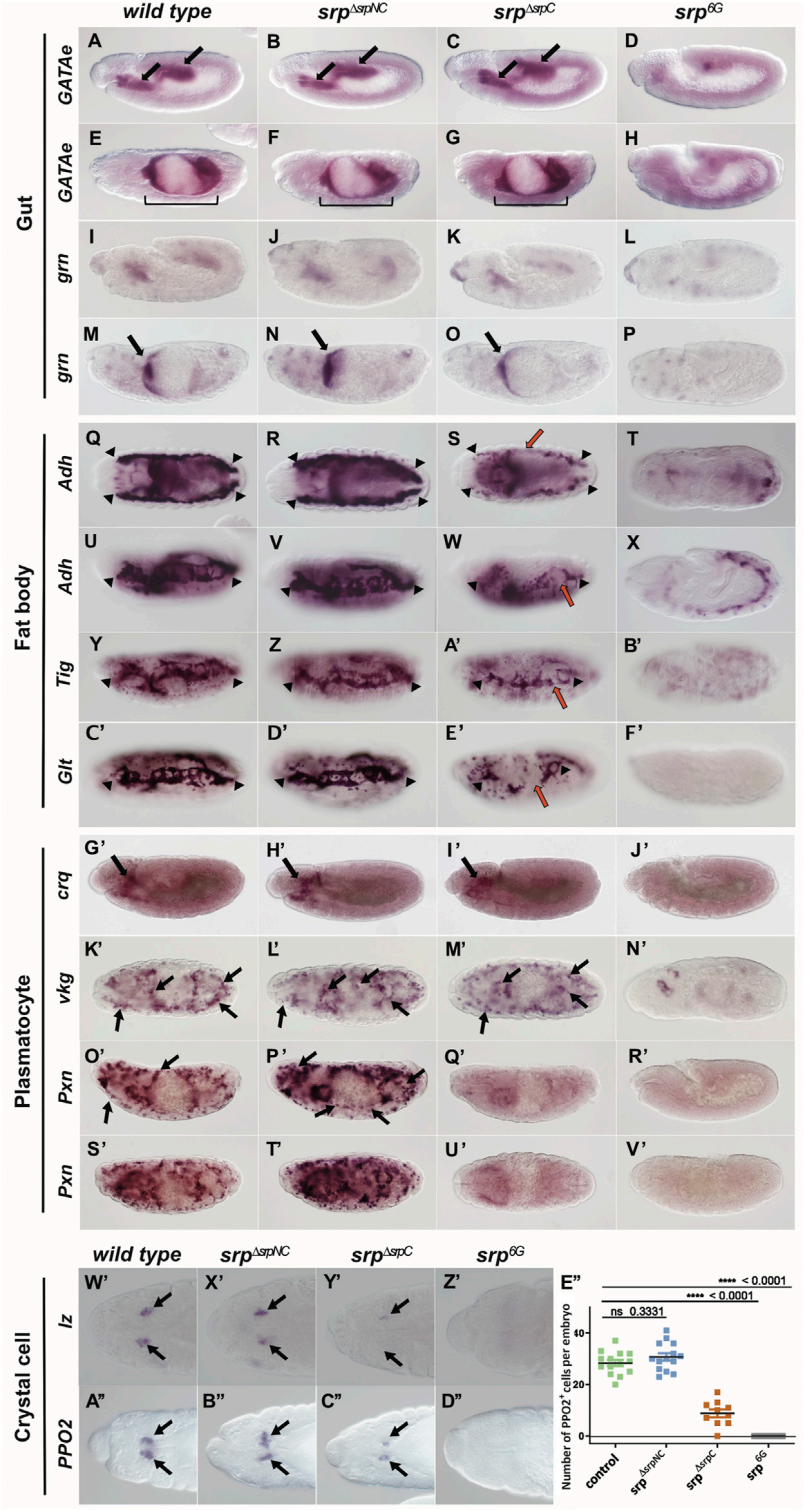
SrpC has essential functions that are only partially compensated by SrpNC during embryonic development. Whole mount *in situ* hybridization of mRNAs expressed in developing gut, *GATAe* **(A–H)** and *grain* (*grn*, **I–P**), mature fat body cells, *Alcohol dehydrogenase* (*Adh*, **Q–X**), *Tiggrin* (*Tig*, **Y–B’**) and *Glutactin* (*Glt*, **C’–F’**), plasmatocytes, *croquemort* (*crq*, **G’–J’**), *viking* (*vkg*, **K’–N’**) and *Peroxidasin* (*Pxn*, **O’–V’**), crystal cells, *lozenge* (*lz*, **W’–Z’**) and *Prophenoloxidase2* (*PPO2*, **A”–D”**) in wild-type, *srp* ^*ΔsrpNC*^, *srp* ^*ΔsrpC*^ and *srp* ^*6G*^ embryos (genotypes are shown on top of each column). Embryonic stages are 12 **(A–D, I–L, G’–J’)**, 13 **(W’–D’’)**, 15 **(E–H, M–P, O’–R’)** and 16 **(Q–F’, K’, N’, S’–V’)**. Although all aspects of gut development (**A–P**; black arrows and brackets) and plasmatocyte development (**G’–V’**; black arrows) are similar in wild-type, *srp* ^*ΔsrpNC*^ and *srp* ^*ΔsrpC*^ conditions, fat body formation (**Q–F’**; delimited by black arrowheads; red arrows point to fat body defects), plasmatocyte specific *Pxn* expression **(O’–V’)** and crystal cell development (**W’–D”**; black arrows) are altered in *srp* ^*ΔsrpC*^ compared to wild-type and *srp* ^*ΔsrpNC*^, but not as much as in *srp* ^*6G*^, where both *srp* isoforms are depleted. **(E’’)** Quantification of *PPO2* expressing crystal cells in wild-type, *srp* ^*ΔsrpNC*^, *srp* ^*ΔsrpC*^ and *srp* ^*6G*^ embryos. *p*-values represent results of a Mann-Whitney test comparing the wild-type with the indicated genotype.

During embryonic development, Srp mediates essential functions in early gut development (Reuter, 1994; Campbell et al., 2011) and in the formation of the fat body, the insect organ analogous to the liver. In *srp* ^*6G*^ loss-of-function mutants, no gut is formed, since for both midgut primordia markers, *GATAe* or *grain*, no expression is detected (Figures 4D,H,L,P). In contrast, neither the loss of *srpNC* (Figures 4B,F,J,N) nor the loss of *srpC* (Figures 4C,G,K,O) are associated to gut developmental defects, and the gut is shaped normally in both mutant contexts. Hence, both isoforms have redundant functions for embryonic gut formation. *srp* ^*6G*^ loss-of-function mutant embryos are also unable to develop mature fat body cells (Rehorn et al., 1996; Sam et al., 1996). Interestingly, loss of *srpC* (*srp* ^*ΔsrpC*^) alters expression of all three fat body markers *Alcohol dehydrogenase* (*Adh*), *Tiggrin* (*Tig*) and *Glutactin* (*Glt*) (Figures 4S,W,A’,E’), compared to wild-type (Figures 4Q,U,Y,C’). Similarly, specific downregulation of *srpC* transcripts by RNAi using the ubiquitous Gal4 driver Tub-Gal4 induces the same fat body alterations as observed for *srp* ^*ΔsrpC*^ mutants (Supplementary Figures S3D–K).

In contrast, in *srp* ^*ΔsrpNC*^ embryos, fat body formation is as wild-type (Figures 4R,V,Z,D’), indicating that SrpNC, but not SrpC, is dispensable for fat body development. However, analyzing *Glt* expression in *srp* ^*ΔsrpC*^/*srp* ^*6G*^ *trans*-heterozygous embryos, reveals a slight stronger fat body defect (Supplementary Figures S4C,G) compared to *srp* ^*ΔsrpC*^ homozygous mutants (Supplementary Figures S4B,F), a phenotype that is closer to the one observed in *srp* ^*6G*^ mutants (Supplementary Figures S4D,H). This little disparity is probably due to the difference in the *srpNC* copy number between the two genotypes and shows that the SrpNC product can only compensate in a very limited way the functions of the SrpC product in this tissue.

### SrpC Isoform Is Required for Embryonic Hematopoiesis

In addition to its function in embryonic gut and fat body development, Srp also plays an essential role in embryonic hematopoiesis (Rehorn et al., 1996). During embryogenesis two blood cell lineages are formed: the plasmatocytes, which are blood cells with phagocytic functions that express the *croquemort* (*crq*) (Franc et al., 1996) and *viking* (*vkg*) (Yasothornsrikul et al., 1997) markers specific for mature phagocytic blood cells, and the crystal cells, the second blood cell type involved in wound healing (Rizki et al., 1980; Lebestky, 2000; Banerjee et al., 2019). In *srp* ^*6G*^ loss-of-function mutants no embryonic blood cells are detected (Figures 4J’,N’) in contrast to *srp* ^*ΔsrpNC*^ (Figures 4H’,L’) and *srp* ^*ΔsrpC*^ mutants (Figures 4I’,M’) in which *crq* and *vkg* expressing plasmatocytes are detected as in wild-type (Figures 4G’,K’). This indicates that SrpNC and SrpC have redundant functions for plasmatocyte formation. However, in *srp* ^*ΔsrpC*^ embryos, expression of another plasmatocyte marker *Peroxidasin* (*Pxn*) (Nelson et al., 1994) is significantly reduced (Figures 4Q’,U’) compared to *srp* ^*ΔsrpNC*^ (Figures 4P’,T’) or wild-type embryos (Figures 4O’,S’), indicating that SrpNC cannot compensate for all SrpC functions during plasmatocyte formation. This specific effect on *Pxn* expression is also observed when *srpC* transcripts are specifically downregulated by RNAi (Supplementary Figures S3L–S). *srp* ^*ΔsrpC*^ mutant embryos also show a significant reduction in the number of crystal cells expressing *lozenge* (*lz*) (Figure 4Y’), the crystal cell fate determinant gene (Lebestky, 2000), and the mature crystal cells expressing the specific differentiation marker *prophenoloxidase 2* (*PPO2*) (Binggeli et al., 2014) (Figures 4C’’,E’’). *Trans*-heterozygote mutant *srp* ^*ΔsrpC*^ with *srp* ^*AS*^, a specific allele of *srp* that abolishes its expression in embryonic hemocytes and not in the fat body (Rehorn et al., 1996), also show a loss of *Pxn* expression in plasmatocytes (Supplementary Figures S4S,W), and a reduced number of crystal cells (Supplementary Figure S4Z) compared to wild-type embryos (Supplementary Figures S4Q,U,Y), showing that defects in the fat body are not responsible for hematopoietic defects. Altogether, our results indicate that during embryonic hematopoiesis the SrpNC isoform is dispensable, while the SrpC isoform is necessary, at least, for *Pxn* expression in plasmatocytes and for crystal cell formation.

### SrpNC Isoform Is Specifically Required to Maintain Larval Blood Cell Homeostasis

**FIGURE 5:**
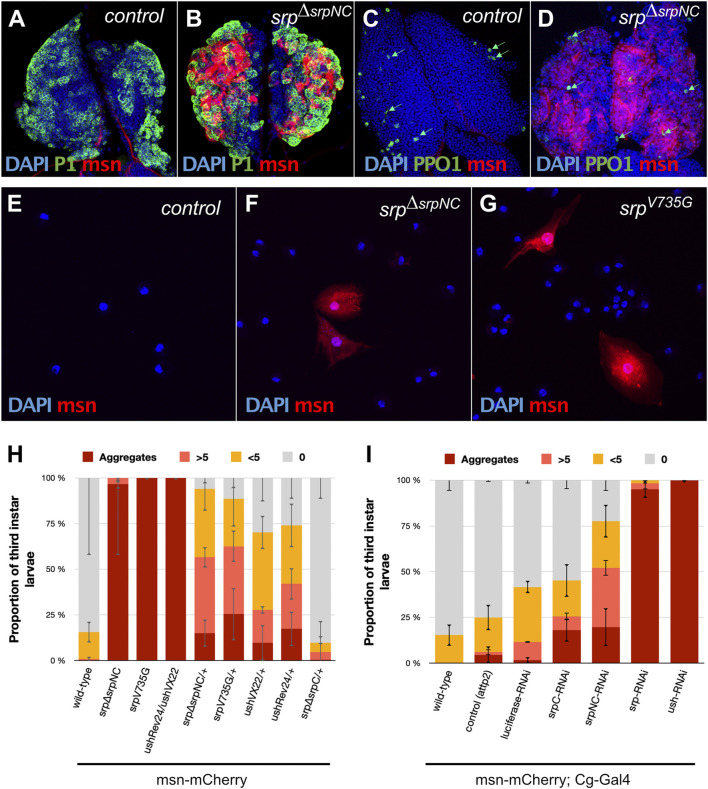
SrpNC/Ush interaction is required to inhibit lamellocyte formation during larval hematopoiesis. Immunostaining against the plasmatocyte marker P1 (green, **(A,B)**), the crystal cell marker *prophenoloxidase 1* (*PPO1*, green, **(C,D)**) and the lamellocyte specific marker (*msn-mCherry*, red, **(A–G)**) in lymph glands **(A–D)** and blood cells of hemolymph of third instar female larvae **(E–G)**. Nuclei are stained with DAPI (blue). Lamellocytes are detected in both lymph glands **(B,D)** and the hemolymph of *srp* ^*ΔsrpNC*^ mutant larvae **(F)** and in *srp* ^*V735G*^ mutant larvae **(G)**. **(H,I)** Hemolymph analysis of third instar larvae using the lamellocyte specific marker *msn-mCherry* (*n* ≥ 40 except for *srp* ^*V735G*^ mutant larvae where *n* = 25). 100% stacked bar charts showing percentage of larvae exhibiting zero (0), very few (<5), a remarkable number (>5) or aggregates of lamellocytes in their hemolymph. Homozygote mutant larvae, as well as the combination of amorphic (*ush* ^*VX22*^) and hypomorphic (*ush* ^*Rev24*^) alleles, display strong lamellocyte production **(H)**. In *srp* ^*ΔsrpNC*^/*+* and *srp* ^*V735G*^/*+* heterozygote larvae, lamellocytes are detected to a lesser extent, except for *srp* ^*ΔsrpC*^/+ that exhibits no significant difference with the wild-type control. Using the *Collagen* driver (*Cg-Gal4*), downregulation of both *srp* isoforms or *ush* leads to massive lamellocyte production. More than 75% of downregulated *srpNC* (*srpNC-*RNAi) shows lamellocyte production, while less than 50% of downregulated *srpC* (*UAS-srpC-*RNAi) does. Error bars correspond to mean deviation.

During larval life, a second wave of hematopoiesis occurs in a specialized organ called the lymph gland (LG) (Jung et al., 2005; Letourneau et al., 2016). Up until the second larval instar, the LG is mostly populated by rapidly dividing blood cell progenitors. At the end of the second larval instar and during the whole third instar, under normal conditions, progenitors start to differentiate into plasmatocytes and crystal cells. However, under stress conditions such as wasp egg infestation, the LG massively produces a third type of hemocyte called lamellocytes, which are essential for wasp egg encapsulation and the formation of melanotic tumors (Rizki and Rizki, 1992; Minakhina and Steward, 2006; Avet-Rochex et al., 2010; Letourneau et al., 2016). Srp plays an essential function in the specification of the hematopoietic fate during *Drosophila* development (Rehorn et al., 1996). From embryogenesis, Srp is highly expressed in the developing LG, in blood-cell progenitors and in all derived hemocytes (Mandal et al., 2004). In *srp* ^*ΔsrpNC*^ mutant larvae, plasmatocytes labelled by the P1 marker (Kurucz et al., 2007) (Figure 5B) and crystal cells expressing the *Prophenoloxidase* 1 (*PPO1*) marker (Dudzic et al., 2015) (Figure 5D) are detected in the LG, as in control lymph glands (Figures 5A,C). However, in contrast to wild-type, in the *srp* ^*ΔsrpNC*^ mutant LG, we observed production of lamellocytes, which express msn-mCherry the lamellocyte specific reporter gene (Tokusumi et al., 2009). Lamellocytes are formed at mid-third instar larval stage even without wasp infestation (Figures 5B,D), and lamellocytes are also detected in the hemolymph of *srp* ^*ΔsrpNC*^ mutant larvae (Figure 5F) and not in control larvae (Figure 5E). Quantification of larvae having lamellocytes in circulation confirms that all *srp* ^*ΔsrpNC*^ mutant larvae display a high content of lamellocytes and aggregates of lamellocytes in their hemolymph (Figure 5H). Moreover, the specific knockdown of *srpNC* by RNAi in hemocytes using the *Collagen-Gal4* (*Cg-Gal4*) driver (Avet-Rochex et al., 2010), leads to a significant production of circulating lamellocytes in the hemolymph (Figure 5I). Altogether, our results demonstrate that SrpNC plays a repressive role on lamellocyte production during larval life. As *srp* ^*ΔsrpC*^ mutant embryos rarely develop until third instar larval stage, we analyzed lamellocyte production in larvae depleted of *srpC* by RNAi as well as in *srp* ^*ΔsrpC*^/+ and *srp* ^*ΔsrpNC*^/+ heterozygous larvae. In *srp* ^*ΔsrpC*^/+ heterozygous larvae only a few lamellocytes are found in their hemolymph, compared to *srp* ^*ΔsrpNC*^/+ larvae, where lamellocytes are present in more than 80% of larvae (Figure 5H). This indicates that in contrast to SrpNC, SrpC is not limiting for inhibition of lamellocyte formation. Nonetheless, downregulation by RNAi of *srpC* using *Cg-Gal4* leads to a mild production of lamellocytes in hemolymph compared to *srpNC* knockdown (Figure 5I). This suggests that SrpC might only slightly contribute to repress lamellocyte formation during larval life. It is noteworthy that we used a *srpC* specific RNAi that reduces efficiently and in a specific manner the expression of the *srpC* isoform in the embryo (see Supplementary Figure S3).

### SrpNC Isoform Is Required During *Drosophila* Oogenesis

**FIGURE 6:**
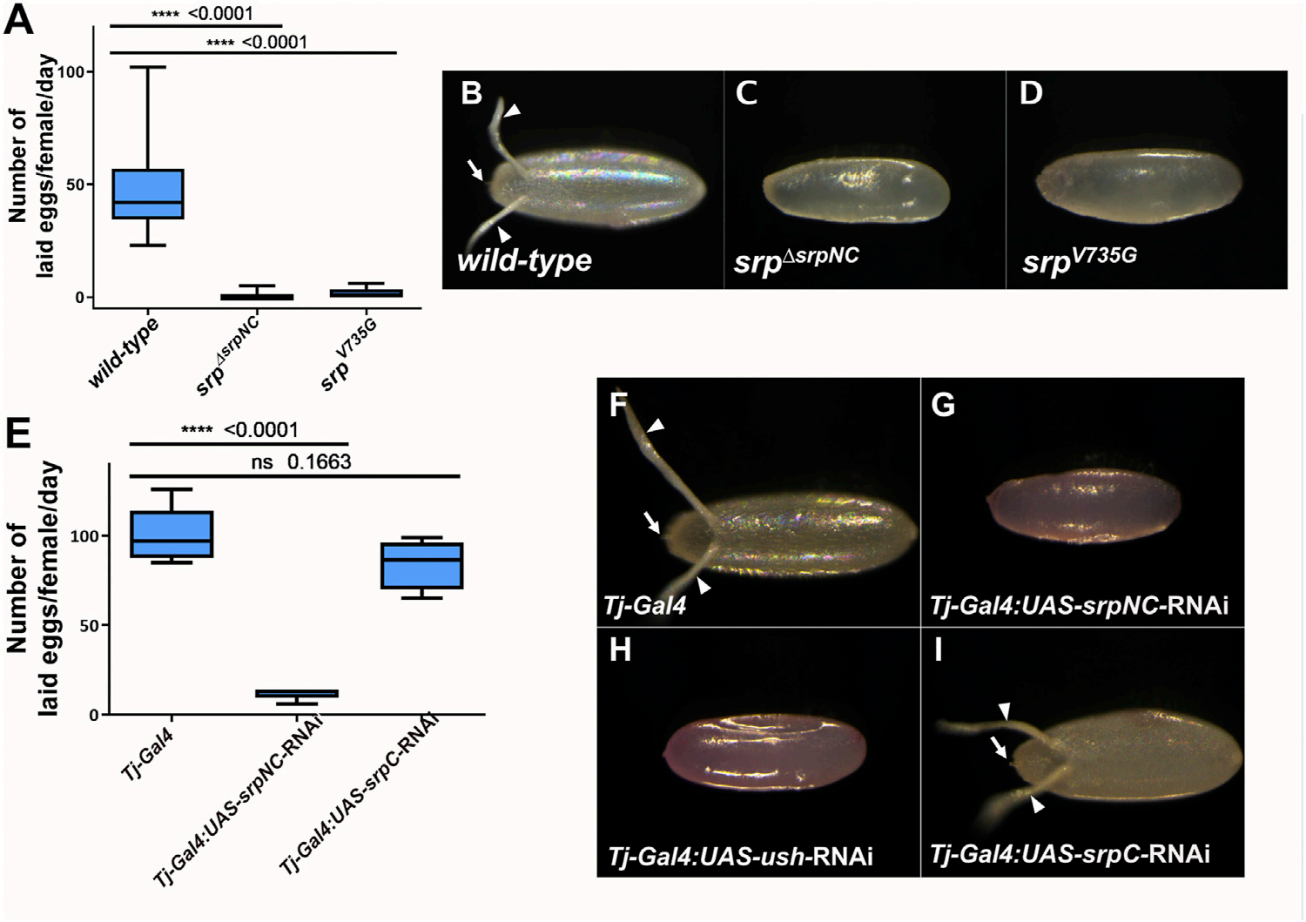
SrpNC/Ush interaction, but not SrpC, is essential for female fertility. Box-whisker plots showing the number **(A,E)**, and morphology **(B–D, F–I)**, of eggs laid by wild-type, *srp* ^*ΔsrpNC*^, *srp* ^*V735G*^ females **(A)** and flies having *srpNC* and *srpC* downregulated by RNAi in the ovarian follicle cells using the *Traffic Jam* (*Tj*) driver (*Tj-Gal4*) **(E)**, at day three after mating. *srp* ^*ΔsrpNC*^ and *srp* ^*V735G*^ flies produce few **(A)** defective **(C,D)** eggs. RNAi downregulation of *srpC* **(E)** in female flies using *Tj-Gal4* does not affect their fertility **(I)** contrary to female knock-down in *srpNC* **(G)** or *ush* **(H)** whose progenies show morphological defects. Unpaired *t*-test was used to compare wild-type **(A)** or background control **(E)** to the indicated genotype.

A recent study shows that *srp* is also expressed and plays essential functions in adult ovaries (Lepesant et al., 2019). The authors show that females in which Srp is depleted by RNAi in ovarian follicle cells, lay almost no eggs, revealing a new role for Srp during oogenesis. We found that although *srp* ^*ΔsrpNC*^ flies develop apparently normally until adult stage, adult female mutants are sterile (Supplementary Figure S5). Furthermore, we found that *srp* ^*ΔsrpNC*^ female flies lay very few eggs compared to wild-type (Figure 6A). Eggs laid by these flies display strong morphological defects (Figure 6C), with an apparently abnormal eggshell and the absence of the dorsal egg respiratory appendages and the micropyle, structures that are produced by the ovarian follicle cells. Females where *srpNC* is downregulated specifically in the ovarian follicle cells using the *traffic jam-Gal4 (Tj-Gal4)* driver (Lepesant et al., 2019), lay similarly defective eggs (Figure 6G). We next asked whether SrpC might also play a role in follicle cells. Interestingly, flies with downregulation of SrpC under the control of Tj-Gal4 lay a number of eggs comparable to control flies. These eggs appear normal (Figures 6E,I) and eventually develop into adult flies (Supplementary Figure S5). This shows that in contrast to SrpNC, SrpC function is dispensable in ovarian follicle cells for egg formation.

### SrpNC Specific Functions Depend on Its Interaction With the FOG Cofactor Ush

To evaluate Ush contributions to SrpNC functions during hematopoiesis and oogenesis in *Drosophila*, we generated and analyzed a new mutant fly line harboring a substitution of this valine to glycine (*srp* ^*V735G*^) that alters its functional interaction with Ush, the *Drosophila* FOG cofactor (Fossett et al., 2003). Analogously to *srp* ^*ΔsrpNC*^ loss-of-function mutants, 75% of *srp* ^*V735G*^ homozygous embryos developed into adult flies (Figure 3). Since we establish that the N-ZnF is dispensable for viability, these results confirm that the interaction of SrpNC with Ush is not critical for fly viability. Our results show also that the SrpNC isoform plays important roles during larval hematopoiesis, and oogenesis. We next asked whether a functional interaction with Ush might be involved for these SrpNC functions. In agreement with previous data (Sorrentino et al., 2007; Avet-Rochex et al., 2010) strong lamellocyte production is detected in the hemolymph of *srp* ^*ΔsrpNC*^, *srp* ^*V735G*^ and *ush* ^*Rev24*^/*ush* ^*VX22*^ mutant larvae (Figures 5F–H). Also, downregulation by RNAi of *srpNC* or of *ush* with *Cg-Gal4* driver augments the production of circulating lamellocytes in the hemolymph compared to control larvae (*UAS-Luciferase-*RNAi) (Figure 5I). Thus, these data suggest that an active functional interaction between SrpNC and Ush is required in blood cells to control lamellocyte formation in larvae. In all four heterozygous *srp* ^*ΔsrpNC*^/+, *srp* ^*V735G*^/+, *ush* ^*Rev24*^/+ and *ush* ^*VX22*^/+ larvae, a significant number of lamellocytes are found in the hemolymph, compared to *srp* ^*ΔsrpC*^/+ or wild-type larvae (Figure 5H), revealing that in contrast to SrpC, SrpNC and Ush are limiting factors for preventing formation of lamellocyte. We next analyzed the contribution of the SrpNC and Ush interaction during oogenesis. Similarly to *srp* ^*ΔsrpNC*^ mutant female flies, we found that *srp* ^*V735G*^ female flies lay only very few eggs (Figure 6A) that display similar defects (Figure 6D) as those produced by *srp* ^*ΔsrpNC*^ mutant females (Figure 6C). In addition, female flies with downregulated *srpNC* or *ush* by RNAi using the *Tj-Gal4* driver also lay similar defective eggs (Figures 6G,H respectively) that do not further develop (Supplementary Figure S5). All these results indicate that the interaction of SrpNC with Ush is also required for normal *Drosophila* egg maturation. Thus, in summary, our results together support the conclusion that all SrpNC specific functions we identified depend on its interaction with its FOG cofactor Ush.

## Discussion

In this study, we investigate the role of two *srp* isoforms during *Drosophila* development. These isoforms are generated by alternative splicing and lead to the production of GATA proteins containing either a single zinc finger, C-ZnF, or two zinc fingers, N- and C-ZnF. Using new mutant flies specific for each isoform, our study reveals that GATA factors with one or two zinc fingers are differentially required in various processes during development and oogenesis.

### The Two Isoforms Perform Mostly Redundant Functions During Embryonic Hematopoiesis

In a previous work, we showed that SrpC and SrpNC differ in their ability to promote gene expression *in vivo* during *Drosophila* embryonic hematopoiesis (Waltzer et al., 2002). Although both isoforms can induce formation *in vivo* of both embryonic blood cell lineages, plasmatocytes and crystal cells, SrpC and SrpNC have specific transactivating capabilities on some of their targets. Ectopic expression of these isoforms in the mesoderm showed that while both isoforms activate the expression of *ush* and *Pxn* with similar efficiency, only SrpC, and not SrpNC, can activate *crq* expression (Waltzer et al., 2002). In this work, we show that in each of the two *srpC* or *srpNC* loss-of-function mutant contexts both embryonic blood lineages are produced, and most marker genes tested are expressed normally, including *crq*. However, in *srpC* loss-of-function mutant embryos we observe alterations in both plasmatocytes and crystal cells. First, we detected a significant reduction in the number of crystal cells and second, we detected one *srp* target gene, *Pxn*, whose expression is strongly reduced in plasmatocytes. Thus, despite their specific properties observed when overexpressed ectopically in the mesoderm (Waltzer et al., 2002), the two isoforms appear to play predominantly, but not fully, redundant functions during embryonic hematopoiesis.

### Incomplete Fat Body Maturation May Compromise Larvae Viability

While the two-fingers isoform (SrpNC), the closest conserved isoform to vertebrates, is dispensable for viability from embryo to adult, the one-finger isoform (SrpC) is essential. The most dramatic phenotype in *srp* ^*ΔsrpC*^ mutant embryos is observed in the fat body, which is not normally formed. *srp* is known to be essential in the early steps of fat body morphogenesis and differentiation, and in *srp* mutant embryos fat body formation is severely compromised (Rehorn et al., 1996). The fat body tissue is a major site for nutrient storage, energy metabolism, innate immunity, and detoxification, and its incomplete development can significantly impair viability (Lemaitre et al., 1996; Senger et al., 2004; Yafei and Yongmei, 2014). Therefore, it is likely that the lethality observed in *srp* ^*ΔsrpC*^ mutant is a consequence of the defect in fat body formation observed in this mutant, although we cannot exclude that additional developmental processes, also affected by the loss of SrpC, could also contribute to *srp* ^*ΔsrpC*^ mutant lethality. In conclusion, at least for fat body formation, the two-fingers isoform SrpNC is largely unable to compensate for the loss of SrpC, establishing that the functions of these two isoforms do not overlap.

### The Specific Functions of SrpNC Involve Interaction With the FOG Factor Through the N-ZnF

Although Srp is required in many essential processes during development, our results show that neither the loss-of-function of SrpNC nor the loss of SrpNC interaction with its FOG cofactor Ush via the N-ZnF affect fly development to adult, establishing that essential functions of Srp are not supported by the N-ZnF domain. This is surprising because phylogenetic analysis shows that SrpNC is the best conserved isoform compared to vertebrate GATA factors. In both mammals and fruit flies, N-ZnF has been shown to be required for interaction with cofactors of the FOG family (Tsang et al., 1998; Crispino et al., 1999). The role of the N-ZnF domain *in vivo* was illustrated in mammals by analysis of mutant mice harboring the V205G substitution in the N-ZnF, which abolishes the physical interaction with FOG cofactors. Introduction of this mutation in GATA1 or in both GATA1 and GATA2, as well as in GATA4, causes embryonic lethality in mice, associated with defects in hematopoiesis or heart formation (Crispino et al., 2001; Shimizu et al., 2004). In *Drosophila*, the corresponding substitution in Srp, V735G, also prevents the functional interaction of SrpNC with Ush. Fossett and colleagues showed that Ush and SrpNC interact to repress crystal cell production, but the co-expression of the non-interacting Srp proteins, SrpNC-V735G with Ush abolishes this repressive effect on supernumerary crystal cells production indicating that, as in mammals, the two proteins must interact to exert their function (Fossett et al., 2003). However, the SrpNC-Ush interaction is not proven to be direct as shown in mammals for GATA1 and FOG by Crispino et al. (Crispino et al., 1999) and remains to be determined by biochemical techniques. Remarkably, the V735G substitution in Srp (*srp* ^*V735G*^) induces similar defects in larval hematopoiesis and oogenesis as the complete deletion of SrpNC. Furthermore, it should be noted that functions of SrpNC that are independent of Ush, are redundant with SrpC functions, such as during embryonic gut formation. Overall, our results support the hypothesis that for the *Drosophila* Srp factor, the primary role of N-ZnF is to functionally interact with its FOG cofactor Ush to allow SrpNC to perform its functions during oogenesis and larval hematopoiesis.

### SrpC and SrpNC Regulate Target Gene Repertoires That Partially Overlap

**FIGURE 7:**
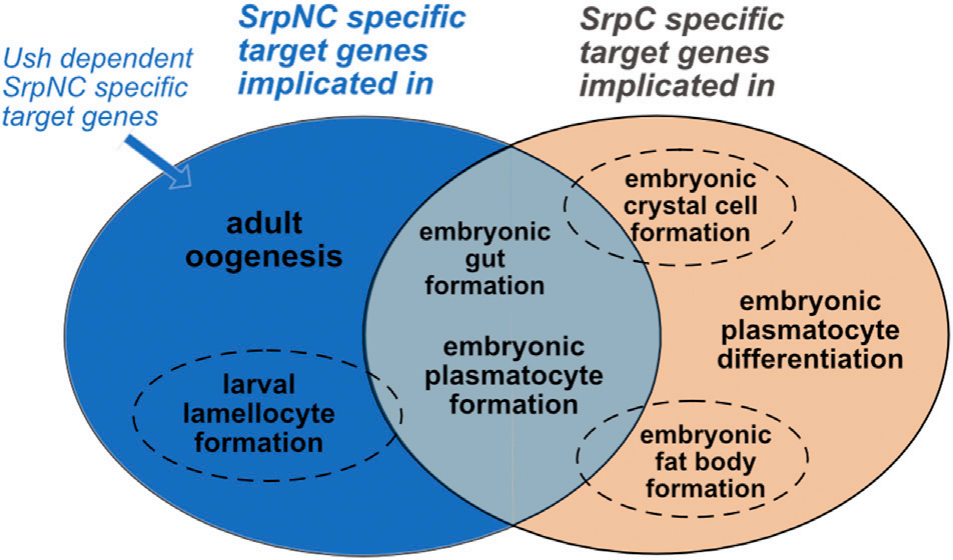
SrpC and SrpNC regulate specific, partially overlapping and completely overlapping repertoires. Gut and plasmatocyte formation are similarly regulated by both SrpNC and SrpC (grey). Crystal cell and fat body development require SrpC and are only partially compensated by SrpNC (dotted circle, orange). In contrast, hematopoietic homeostasis (reflected by lamellocyte formation inhibition) depends mainly on SrpNC and might only slightly be dependent on SrpC (dotted circle, blue). Both isoforms have specific functions: SrpNC controls oogenesis (blue) and SrpC at least partially controls plasmatocyte differentiation (as shown by *Pxn* dependence on SrpC expression in plasmatocytes, orange). All currently identified SrpNC mediated functions are dependent of its interaction with Ush.

Our results show that depending on the tissue and developmental stage, the two isoforms have specific functions. During embryonic gut and plasmatocyte formation, the loss of either isoform has no effect, indicating that there is complete functional redundancy between SrpC and SrpNC. This does not appear to be the case during fat body and crystal cell formation, as well as for plasmatocyte maturation, where the two-fingers isoform SrpNC is unable to fully compensate the loss of SrpC, indicating that the functions of these two isoforms only partly overlap. Furthermore, the Srp specific functions that rely on its interaction with Ush, like inhibition of lamellocytes production or eggs formation, depend specifically on SrpNC and are not compensated by SrpC. Of note, in a previous work we show that the presence of the N-ZnF in Srp stabilizes binding to double palindromic GATA sites (Waltzer et al., 2002), suggesting that SrpNC, and not SrpC, might regulate a specific target gene repertoire with such type of GATA binding sites in their regulatory region. Surprisingly, we have not identified such a specific function for SrpNC, not redundant with SrpC, and that is Ush-independent. This suggests that *in vivo*, despite its strong evolutionary conservation, the N-ZnF of Srp does not provide any additional stability for more complex sites as previously proposed (Trainor et al., 1996). Interestingly, allelic combinations of *srp* ^*ΔsrpC*^ with *srp* ^*3*^, a *srp* allele that contains a point mutation in the C-Znf preventing the binding of both isoforms to DNA, shows a strong loss of viability phenotype like the one observed with the loss-of-function allele *srp* ^*6G*^. This suggests that the binding to their target genes is necessary to achieve their functions, and therefore that the two isoforms would only have partially overlapping target gene repertoires. In conclusion, our analysis leads us to divide Srp functions into three categories of target gene repertoires (Figure 7): 1) target genes for which the presence of the C-ZnF is sufficient, SrpNC and SrpC regulating them redundantly (Figure 7, grey); 2) target genes specifically assigned to the SrpC isoform: these are genes for which SrpNC, despite the fact that it also contains the C-ZnF, cannot compensate the loss of SrpC (Figure 7, orange); 3) finally, the third category corresponds to target genes whose regulation requires the presence of both N- and C-ZnF, and presumably also requires the interaction with the FOG cofactor Ush (Figure 7, blue). Thus, our study establishes that each isoform has distinct roles during *Drosophila* development.

### An Alternate Exon to Escape the FOG Cofactor Interaction

The GATA family is evolutionarily conserved and has expanded in many animal lineages, six GATA genes have been identified in many vertebrates, five in many insects, and six to fourteen in nematodes. Most metazoan GATA factors possess dual zinc fingers, and there is considerable evidence for an ancestral GATA gene common to vertebrates and invertebrates that encoded the two-fingered product (Gillis et al., 2009; Eurmsirilerd and Maduro, 2020). Furthermore, most probably the last common protostome/deuterostome ancestor had at least two distinct classes of GATA factors, GATA123 and GATA456 (Gillis et al., 2008; Gillis et al., 2009). While expansion of vertebrate GATA gene families occurred mainly *via* genome duplication events, in protostome, including *Drosophila*, expansion occurred by tandem duplications from an ancestral GATA456 gene, a group to which *srp* belongs. Invertebrate GATA factors that possess only a single zinc finger all arose from two-fingered ancestral sequences (Gillis et al., 2009). In the case of *srp*, the *srpC* product probably appeared after a duplication of the exon encoding the N-ZnF, associated to an original mutually exclusive splicing mechanism (Yue et al., 2016). Since we fail to find evidence of an alternative exon in other branches of arthropods whose genomes are sequenced, the alternative splicing mechanism leading to the production of the SrpC isoform probably appeared in hexapods such as the Collembola *Folsomia candida*, before appearance of the insects.

Both zinc finger domains as well as critical amino acids, like those required for the interaction between GATA and FOG factors, are well conserved from human to *Drosophila*. In *Drosophila* larval hematopoiesis and adult oogenesis, Srp function relies on the SrpNC isoform and its ability to interact with the FOG cofactor Ush. As both mutants, *srp* ^*ΔsrpNC*^ and *srp* ^*V735G*^, share the same phenotype, it is tempting to speculate that the emergence of this alternate exon (E4B in *Drosophila*) allows Srp to avoid interaction with its FOG cofactor Ush, thus extending the range of regulatory options of the GATA factor.

### Different Evolutionary Route Followed by Invertebrates and Vertebrates GATA Transcription Factors

In vertebrates, no alternative splicing strategy has emerged so far and only GATA factors with two zinc fingers are found. Studies carried out with the mouse GATA1 have shown that, on one hand, C-ZnF is necessary for motif recognition and DNA binding, and that, on the other hand, N-ZnF contributes to the specificity and stability of DNA binding on more complex binding sites. However, transgenic rescue experiments conducted with the GATA1 mutant mouse revealed that N-ZnF is required for definitive erythropoiesis but dispensable for primitive erythropoiesis, illustrating that, depending on the context, the two zinc finger domains are not required for specific GATA factor functions (Shimizu et al., 2001). Here we show that the two Srp isoforms indeed have distinct but also common functions during *Drosophila* development. The fact that the presence of either SrpC or SrpNC can compensate for the lack of one member indicates that they have redundant roles and that the presence of the N-ZnF domain is not required to regulate common targets implicated in gut development or early embryonic hematopoiesis. Also, the conserved feature of Srp with GATA4 and GATA6 in mammals, as an inducer of an epithelial to non-polarized migratory cell transition, also called EMT, is a function that is entirely supported by SrpC or SrpNC in *Drosophila*, while this function is carried by these two-fingered mammalian GATA factors (Campbell et al., 2011) suggesting here too that the N-ZnF does not play any important function. As mentioned above, the loss of a zinc finger could have led to the expansion of the Srp target repertoire, and indeed, our study establishes that there are also SrpC specific targets that cannot be regulated by SrpNC. This suggests that *in vivo*, the N-ZnF restricts rather than extends the ability of GATA factors to regulate the repertoire of C-ZnF bound target genes. Thus, in addition to gene duplication, alternative splicing is also an effective strategy for promoting sub- and neo-functionalization.

In conclusion, our work sheds further light on the versatile mode of action of GATA transcription factors by revealing an unexpected mode of action *in vivo* for a GATA factor where the N-terminal finger does not bring any additional binding capacity as previously thought but instead plays a restrictive role in the selection of target genes. They also open the road to the characterization of the molecular mechanisms at the basis on this selectivity in specific developmental or pathological contexts.

## Materials and Methods

### Fly Strains

All *Drosophila melanogaster* stocks, and crosses were maintained using standard medium at 25°C. The fly strains were, *srp* ^*3*^ (BL2485), *srp* ^*AS*^ (BL59020), *Tub-Gal4* (BL5138), *w* ^*1118*^ (used as wild type background, BL3605), *attP2* (BL25710) from the Bloomington *Drosophila* Stock Center, UAS-dsUsh (GD5712) (from Vienna *Drosophila* Resource Center) and *srp* ^*6G*^ (Reuter, 1994). The strains *ush* ^*VX22*^ and *ush* ^*Rev24*^ were supplied by P. Heitzler. The fly lines *msn-mCherry* and *Tj-Gal4* were kindly provided by the R. Schulz lab and Luisa Di Stefano, respectively.

### Generation of Mutant Fly Strains by CRISPR/Cas9 Genome Editing System

For *srp* ^*∆srpNC*^ and *srp* ^*∆srpC*^ mutant fly lines two different single guide RNAs (sgRNAs) were used, and for the *srp* ^*V735G*^ fly line one guide and a single-strand DNA donor (ssDNA) of 200 base pairs harboring the mutated nucleotides (GGA to GTC) were used. The zero-off-target-sites, sgRNAs and the ssODN donor were designed according to the protocol on the fly CRISPR website https://flycrispr.org/(Gratz et al., 2014) and produced after cloning using the pCFD3 plasmid (addgene #49410) as described on https://www.crisprflydesign.org/(Port et al., 2014). Plasmid injection was performed at a concentration of 250 ng/μL for sgRNAs and 100 ng/μL for ssODN. DNA preparations were injected into embryos expressing the nuclease Cas9 in germline cells under the control of the *vasa* promoter (Bloomington stock BL51323). Screening for mutations was done by PCR and identified mutated alleles were sequenced for validation (Supplementary File 1A). Sequences of gRNAs, ssODN, primers used for screening and for sequencing are provided in the Supplementary File 1B.

### Generation of Transgenic RNAi fly Lines

RNAi constructs were designed using the E-RNAi web service. 21-nucleotide sequences of targeting regions of *srp* exon 4A or 4B specific for srpNC and srpC were chosen, respectively. Sequences with the lowest off-target score were selected and blasted, using the National Center for Biotechnology and Information (NCBI) website, against the *D. melanogaster* RNA-sequences available at the NCBI Reference RNA Sequences (Refseq_rna) database, in order to validate the absence of matches with off-target sites. Short-hairpin RNAs (shRNAs) were designed as described in (Ni et al., 2011) and synthetized by the Integrated DNA Technologies platform. For each shRNA construct, synthetized sense and anti-sense strands were annealed and cloned into the pWalium20 plasmid (DGRC: 1472), following a protocol adapted from the cloning in pCFD3 vector protocol (https://www.crisprflydesign.org/). Recombinant plasmids were individually injected into flies containing attp2 sequence in the genome and expressing the φC31 integrase under the control of *nanos* (BL25710). shRNA sequences are provided in the Supplementary File 1B.

### Reverse-Tanscription Polymerase Chain Reaction and Quantitative RT-Polymerase Chain Reaction

For Reverse-transcription polymerase chain reaction (RT-PCR) in Figure 2A, flies of the *w* ^*1118*^ genotype were allowed to develop at 25°C and tissues from these flies were dissected in 1x phosphate buffered saline (PBS). Total RNA was extracted using the RNeasy Plus Mini kit (Qiagen). RT was performed using random primers (*Invitrogen*, P/N 58875) and SuperScript™ II Reverse transcriptase kit, and the PCR was done using GoTaq DNA polymerase (Promega). See Supplementary File 1B for the primer sequences. For RT-PCR (Supplementary Figure S1C, D) and quantitative RT-PCR (qRT-PCR), embryos were allowed to develop until stages 14–16 on agar plates, at 25°C. RT was done as described above, while qPCR was performed using a CFX Connect real-time PCR detection system (Bio-Rad) and EvaGreen (Bio-Rad). All samples were analyzed in triplicates, and quantification was performed using the comparative threshold cycle (*ΔΔCT*) method as described by the manufacturer (CFX Maestro Software). *rp49*, *Act42A*, *RPL32* and *RPS20* were used as a normalization control, and graphs representing RT-qPCR data contain averages and standard deviations and the *p*-value is calculated using an unpaired *t*-test. Primers used are listed in the Supplementary File 1B.

### Survival Analysis

Throughout the survival analysis period, flies were raised at 25°C. For each analyzed genotype, embryos at stages 14–16 were collected on agar plates and their ability to hatch was recorded. 48 h later, the ability of the developed first instar larvae to reach third instar larval stage was quantified, and third instar larvae (L3) were transferred to tubes containing standard media, where analysis of their ability to develop into pupal and adult flies was performed 48 h and 5 days after the L3 transfer, respectively.

### *In Situ* Hybridization

*In situ* hybridizations were carried out as described previously using a Dig-UTP or fluorescein-UTP-labelled antisense RNA probe (Waltzer et al., 2002). RNA probes specific to the transcript of genes *Alcohol dehydrogenase*, *croquemort*, *GATAe*, *Glutactin*, *grain*, *lozenge*, *Prophenoloxidase 2*, *Peroxidasin*, *Tiggrin* and *viking* were obtained from corresponding cDNA plasmids from the *Drosophila* Golden Collection. Images were acquired using the Nikon Eclipse 80i microscope and the NIS-Element software and were assembled using ImageJ.

### Immunostaining

Lymph glands were dissected and processed as previously described (Louradour et al., 2017). For immunofluorescent staining on circulating cells, four female third instar larvae were bled in 1 ml of PBS in 24-well-plate containing a glass coverslip. Hemocytes were centrifuged for 2 min at 900 g, fixed for 20 min with 4% paraformaldehyde in PBS and washed twice in PBS. Cells were then permeabilized in PBS0.3% Triton (PBST), blocked in PBST-1% Bovine Serum Albumin (BSA) and incubated with primary antibodies at 4°C over night in PBST-BSA. Next, cells were washed in PBST, incubated for 2 h at room temperature with corresponding Alexa Fluor-labeled secondary antibodies (Molecular Probes), washed in PBST and mounted in Vectashield medium (Eurobio-Vector) following incubation with DAPI. Imaging was performed on a Leica SP8 confocal microscope. All analyzed larvae are females having one copy of the X-linked transgene *msn-mCherry*. Primary antibodies were mouse α-P1 (1/30), and rabbit α-PPO1 (1/10,000). The P1 antibody were provided by M. Crozatier and PPO1 from (Li et al., 2012). Confocal sections were acquired on a Leica SP8 microscope and were assembled using ImageJ. For each experiment, images of different genotypes were taken with the same signal intensity level.

### Quantification of Lamellocyte and Categories Classification

Live larvae were observed on a Leica fluorescence detector macroscope and were classified into four categories based on the number of circulating cells expressing msnF9-mCherry detected in their hemolymph. Larvae without fluorescent circulating cells in the hemolymph were classified as “0”. Larvae with 1-5 fluorescent cells were classified as “<5”. The presence of a high number of msnF9-mCherry expressing cells was classified as the category “>5 lamellocytes” and larvae with clusters of mCherry expressing cells were classified as larvae with “aggregates” of lamellocytes.

### Analysis of the Fertility Phenotype

Virgin female flies were crossed to *w^1118^* males with a ratio of two males per female and putted on agar plates at 25°C. The number of laid eggs was quantified 3 days after the initial cross. Photos of the laid eggs were taken using a Leica macroscope. For quantification of adult progenies, at least three tubes containing five females of each genotype of interest crossed to three *w* ^*1118*^ males, were placed at 25°C, and emerged adult progenies (F1 generation) were counted.

### Database Search

To find all potential Srp homologs in Arthropods, we used the NCBI Resource Center blastp site (https://blast.ncbi.nlm.nih.gov/). The closest Srp homologs from *Drosophila melanogaster* were used in several independent iterative PSI-BLAST searches against all available predicted protein databases in NCBI. The corresponding alternative exon of each gene was identified, by using as a query the different alternative exons already identified, always positioned between the two exons coding for the N- and C-Zn finger. References for the presented sequences in Figure 1 are given in the Supplementary File 1C.

## Data Availability

The original contributions presented in the study are included in the article/Supplementary Material, further inquiries can be directed to the corresponding author.
