# Supplementary figures and images for: Two Isoforms of serpent Containing Either One or Two GATA Zinc Fingers Provide Functional Diversity During Drosophila Development
Source: Front Cell Dev Biol. 2022 Feb 1;9:795680. doi: 10.3389/fcell.2021.795680 (PMC8844375; doi:10.3389/fcell.2021.795680)

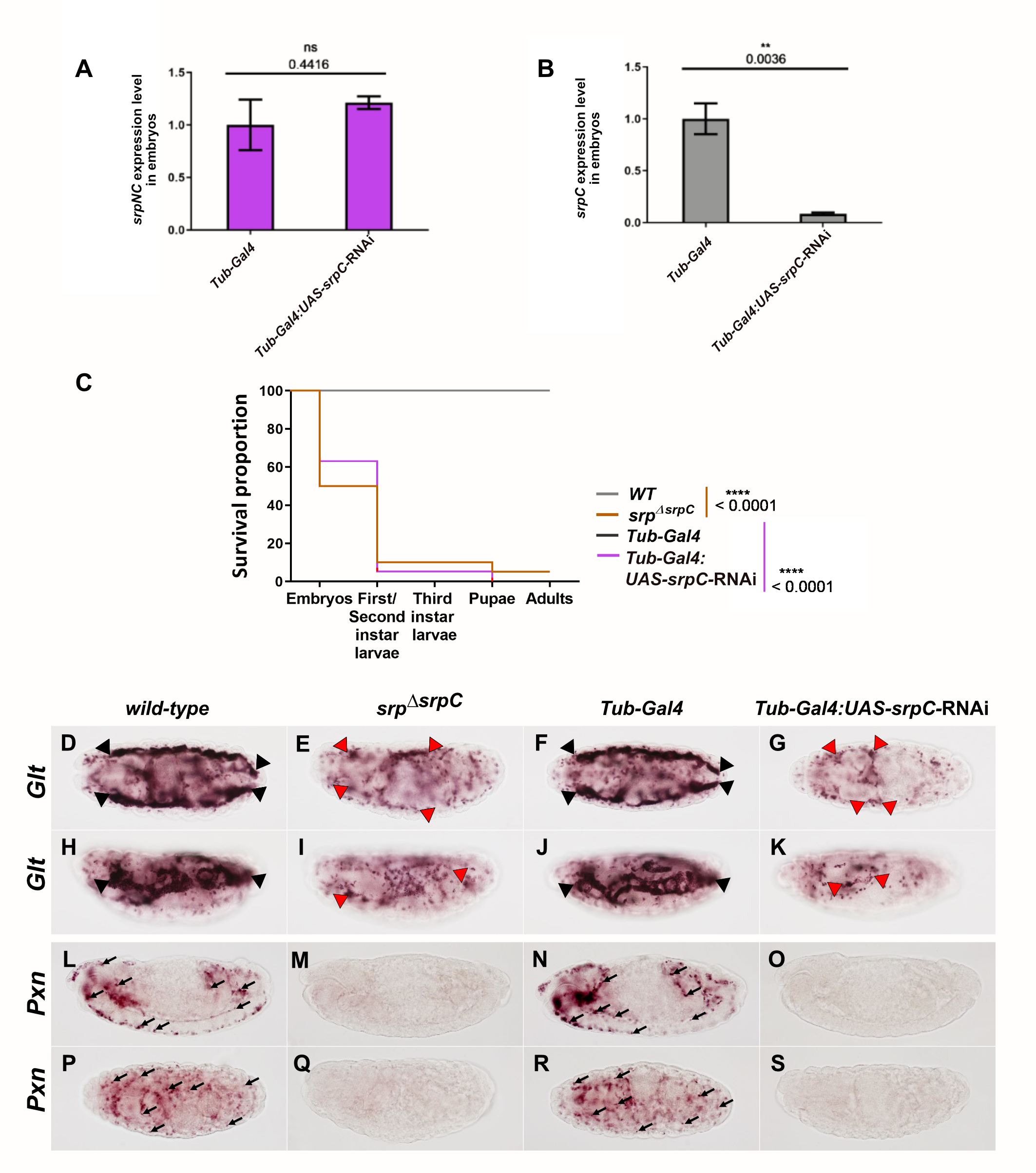

Supplement: Supplementary file 1 [file Image3.tiff]

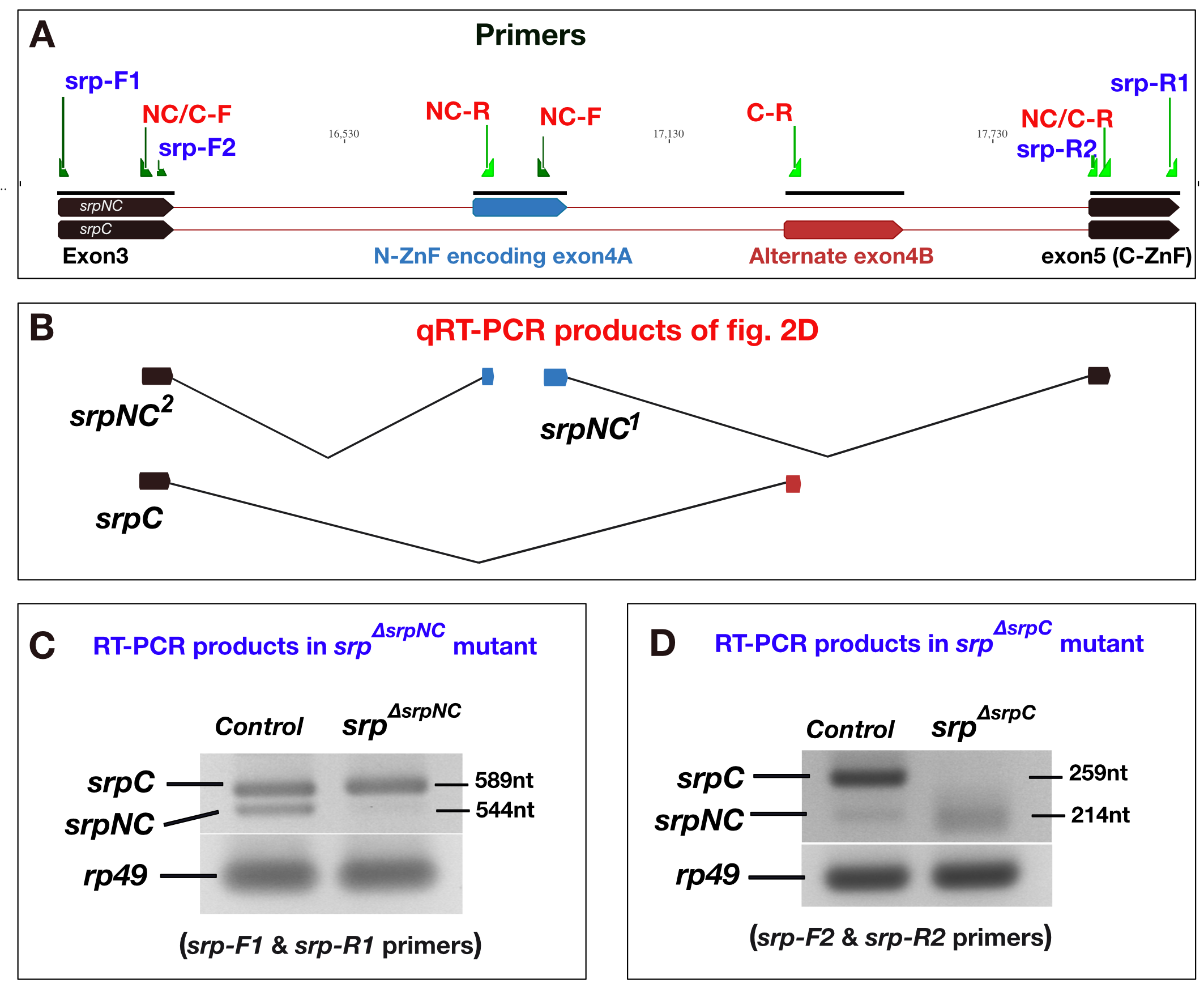

Supplement: Supplementary file 2 [file Image1.tiff]

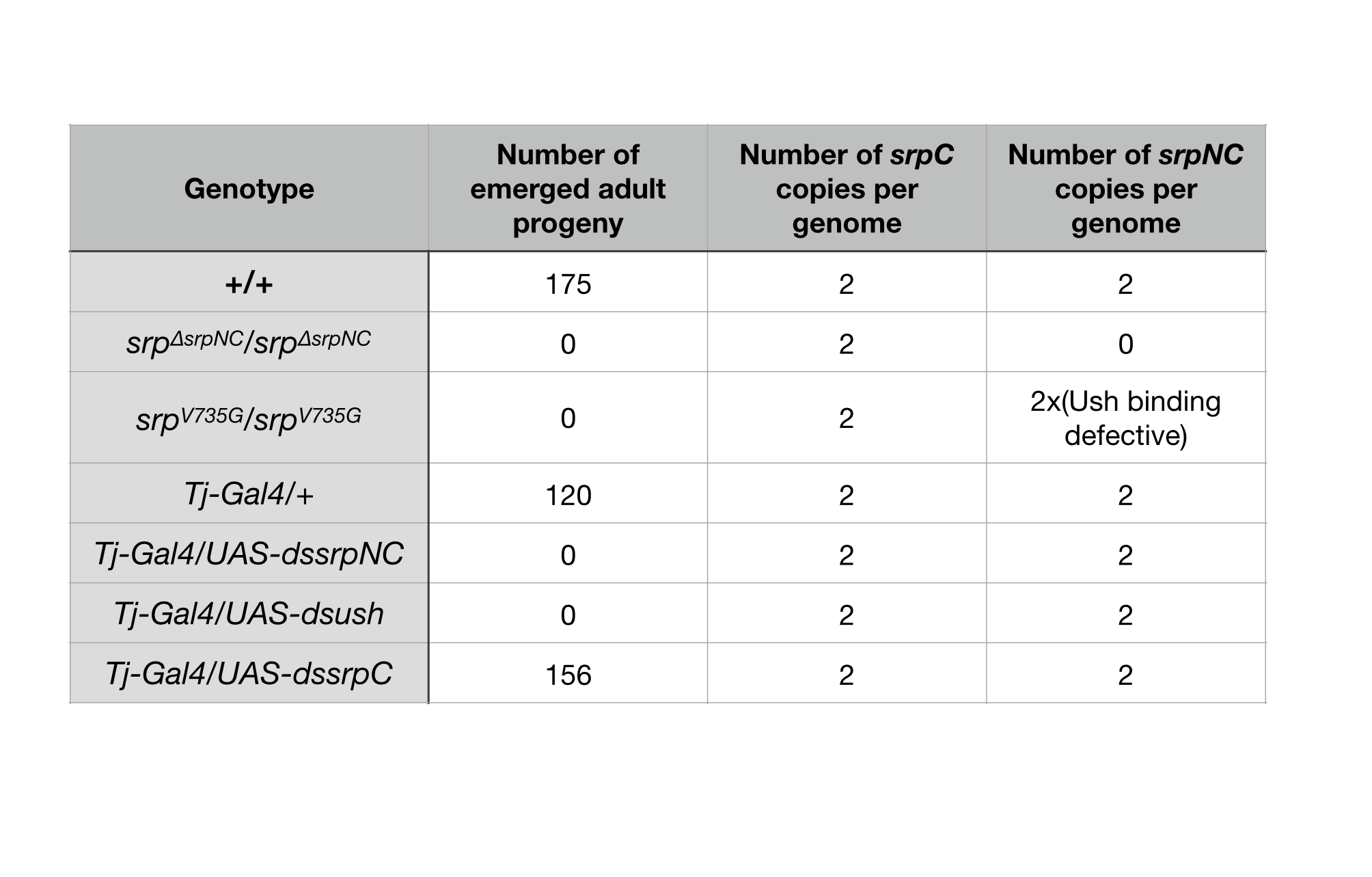

Supplement: Supplementary file 3 [file Image5.tiff]

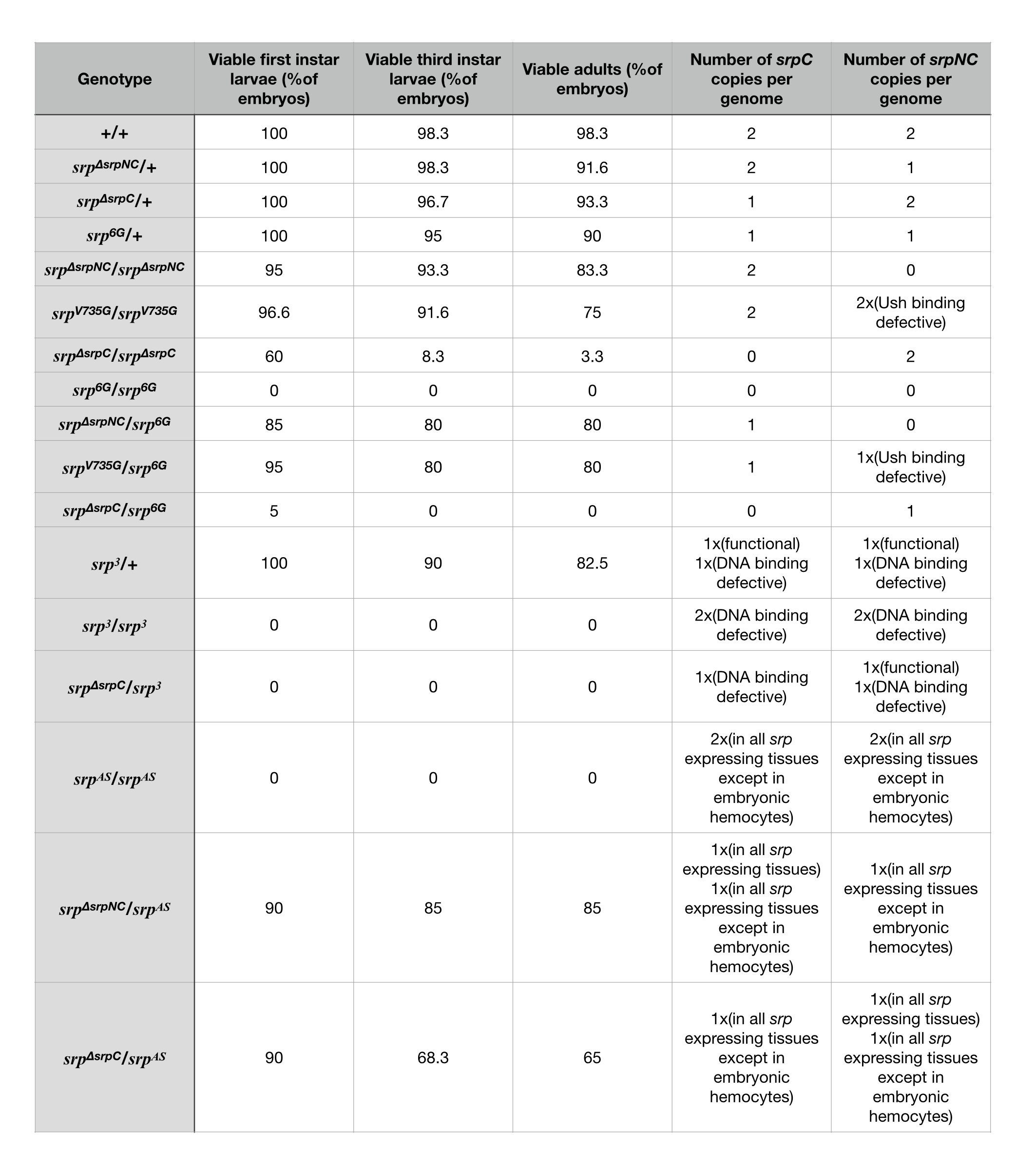

Supplement: Supplementary file 5 [file Image2.tiff]

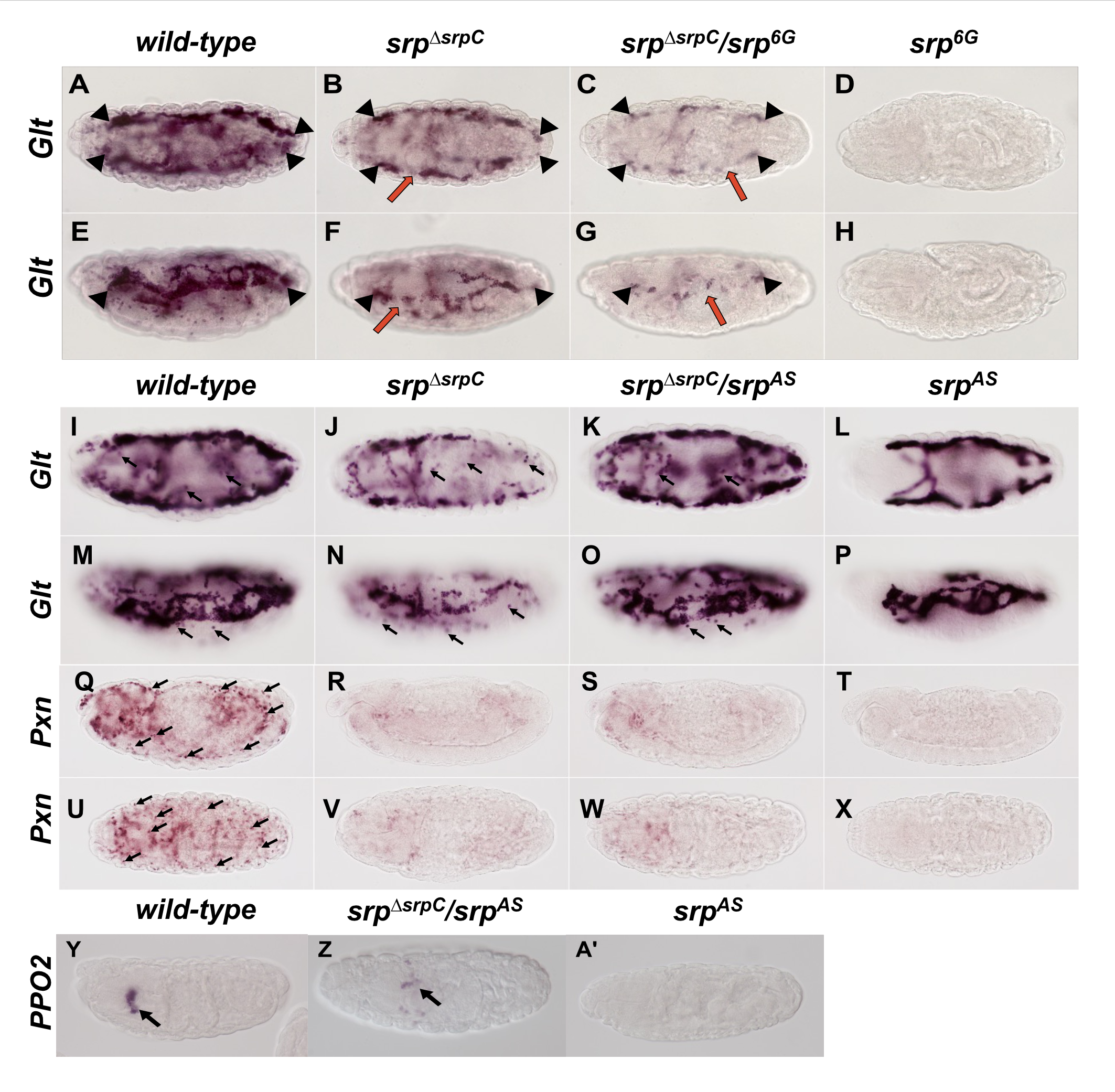

Supplement: Supplementary file 6 [file Image4.tiff]
