# Supplementary material for: Two Isoforms of serpent Containing Either One or Two GATA Zinc Fingers Provide Functional Diversity During Drosophila Development
Source: Front Cell Dev Biol. 2022 Feb 1;9:795680. doi: 10.3389/fcell.2021.795680 (PMC8844375; doi:10.3389/fcell.2021.795680)
Supplement: Supplementary file 4 [file DataSheet2.docx]

**Supplemental file 1**

**A- Results of mutated alleles sequencing**

***srp^∆srpC^*** CGGCATGAATCGACCCCTAATTAAACAGCCTAGAAGATTGGTAAATGTCTAAAACAAATTCACAAAATCACAAAACTACCACCAGCAATTACTACTAACCAATACCAATCAATCAAAACAAACGCTTTTGTGTGGAACAAAATTTCTGTGTGCAACATCCTCTCTCAGTGTTTAGTGGGTAGTGGGTACTCGTAACTTATAGAAATCCGCATCCGCCAGCATCAGCACCACCGCCCCACCTTCACCCAGCCCCCCCCCCTGAAAAGCTCCAATGCTCCAAGANAGTGAAAAAATTAATTGAATTTATTATTTATTACGCAATGCCAACTGCTGGTGCCGCCAGCTCCTACTCCTGTCCAGGCTCGAATGCAACCTCGGCGGCGACTTCGGCGGTGGCGTCGGGAACGGCAGCAACTGCAGCGACNACGCTCGACNAGCACGTTAGTCGCGCCAATTCNAGACGCTTGGTAGGTCGCCCATTAATTACCGATACCGATACCGAAACCAAAAACCAAACCAAATCAAACAAAACCAACCAAACCAAACCCATACCAAAACCCTACCANCANACCGAAACCCTTTTGAATAGAAAAAACGCACTA

***srp^∆srpNC^***CCAAATCAACTCAAATCAAACANAACACCTAGCAGATTGGTAAATGTCTAAAACAAATTCACAAAATCACAAAACTACCACCAGCAATTACNACTAACCAATACNAATCAATCAAAACAAACGCTTTTGTGTGGAACAAAATTTCTGTGTGCAACATCCTCTCTC

***Srp^V735G^***

TTATTCGATGCCGATTATTTTACAGAAGGACGTGAGTGTGGAAACTGTGGTGCGATTTCAACCCCATTATGGCGACGCGATAACACGGGACACTATTTGTGCAATGCCTGCGGCTTGTACATGAAAATGAACGGCATG

**B- List of oligos**

*srp^∆srpNC^* sgRNA: ATAAGTCATAAGGTTTTGCT

*srp^∆srpNC^* sgRNA: AATTAAACAGCCTAGAAGAT

*srp^∆srpC^* sgRNA: CACTTTTCGATTAAACTAGT

*srp^∆srpC^* sgRNA: CGTAGTAAGGCTAACACGAG

*srp^V735G^* sgRNA: ATGCCGATTATTTCACTGAG

*srp^∆srpNC^* screeningForward: AATTCAAACCAACAAAAAGACACCT

*srp^∆srpNC^* screeningReverse: GCAGATGCAGATTGATCAGATTTTC

*srp^∆srpC^* screeningForward: CCCCTGAAAAGCTCCAATGC

*srp^∆srpC^* screeningReverse: CTCAGTGGCCAAGGAGGTTT

*srp^V735G^* screening Forward: ACACAATACGCAAATGC

*srp^V735G^* screening Rm: CAGTTTCCACACTCACG

*srp^V735G^* screening Reverse: ACTGAGAGAGGATGTTGC

*srp^∆srpNC^* sequencing: ACACACACAATACGCAAATGCA

*srp^∆srpC^* sequencing: GCGATTTCAACCCCATTATGG

*srp^V735G^* sequencing: ACACACACAATACGCAAATGC

*srp1* RT-PCR Forward: GATACCTGGTTCGATCC (called: srp-F1)

*srp1* RT-PCR Reverse: TGGTGTCCTTTTTCATG (called: srp-R1)

*srp2* RT-PCR Forward: CTCGGCATCGTTGTC (called: srp-F2)

*srp2* RT-PCR Reverse: TCCGGCTCGCTTTGAGG (called: srp-R2)

common *srpC* and *srpNC* Forward (qRT-PCR): GAACCACTGGACGCGTAGAT (called: NC-F)

common *srpC* and *srpNC* Reverse (qRT-PCR): GCAGTTGGAGCAGGAAAGTC (called: NC/C-R)

*srpC* specific Reverse (qRT-PCR): ATAGAAATCCCCGCCTGATT (called: C-R)

*srpNC* specific Forward (qRT-PCR): CATGAAAATGAACGGCATGA (called: NC-F)

*srpNC* specific Reverse (qRT-PCR): CTCGCCCCTCAGTGAAATAA (called: NC-R)

*srp* Forward (qRT-PCR): AACAACAGCTCGATCTTCAACA

*srp* Reverse (qRT-PCR): TCAGTTGCTGAGCTTGCAGA

rp49 Forward (qRT-PCR): GACGCTTCAAGGGACAGTATCTG

rp49 Reverse (qRT-PCR): AAACGCGGTTCTGCATGAG

RpL32 Forward (qRT-PCR): ATGCTAAGCTGTCGCACAAATG

RpL32 Reverse (qRT-PCR): GTTCGATCCGTAACCGATGT

RpS20 Forward (qRT-PCR): TGTGGTGAGGGTTCCAAGAC

RpS20 Reverse (qRT-PCR): GACGATCTCAGAGGGCGAGT

Act42A Forward (qRT-PCR): GCGTCGGTCAATTCAATCTT

Act42A Reverse (qRT-PCR): AAGCTGCAACCTCTTCGTCA

exon 4A specific shRNA sense: CTAGCAGTGCATGAATCGACCCCTAATTATAGTTATATTCAAGCATATAATTAGGGGTCGATTCATGCGCG

exon 4A specific shRNA antisense: AATTCGCGCATGAATCGACCCCTAATTATATGCTTGAATATAACTATAATTAGGGGTCGATTCATGCACTG

exon 4B specific shRNA sense: CTAGCAGTCTATAAACCCAACTCATTTAATAGTTATATTCAAGCATATTAAATGAGTTGGGTTTATAGGCG

exon 4B specific shRNA antisense:

AATTCGCCTATAAACCCAACTCATTTAATATGCTTGAATATAACTATTAAATGAGTTGGGTTTATAGACTG

*srp^V735G^* ssDNA sequence:

CGTTCATTTTCATGTACAAGCCGCAGGCATTGCACAAATAGTGTCCCGTGTTATCGCGTCGCCATAATGGGGTTGAAATCGCACCACAGTTTCCACACTCACGTCCTTCTGTAAAATAATCGGCATCGAATAATGCTTCAAAGATTGACATAGAAATATCGGAATAAGTCATAAGGTTTTGCTAGGTGTTTGTTTGATT

**C- References of Srp homologs in Arthropods**

**Diptera**

*Drosophila melanogaster*: NT_033777.3; *Rhagoletis zephyria*: LOC108362397; *Musca domestica*: LOC101899376;

**Siphonaptera**

*Ctenocephalides felis*:LOC113370855;

**Hymenoptera**

*Bombus bifarius*: LOC117209408; *Temnothorax curvispinosus*: LOC112452452;

**Coleoptera**

*Tribolium castaneaum*: LOC661909;

**Thysanoptera**

*Frankliniella occidentalis*: LOC113211906;

**Hemiptera**

*Nilaparvata lugens*: LOC111056291;

**Psocodea**

*Pediculus humanus corporis*: Phum_PHUM490230_-_8235735;

**Dyctyoptera**

*Zootermopsis nevadensis*: LOC110831978;

**Palaeoptera**

*Cloeon dipterum*: CADEPI010000456.1;

**Hexapoda, Collembola**

*Folsomia candida*: LOC110845967 - 110845967.
