# Supplementary material for: Two Isoforms of serpent Containing Either One or Two GATA Zinc Fingers Provide Functional Diversity During Drosophila Development
Source: Front Cell Dev Biol. 2022 Feb 1;9:795680. doi: 10.3389/fcell.2021.795680 (PMC8844375; doi:10.3389/fcell.2021.795680)
Supplement: Supplementary file 7 [file DataSheet1.docx]

**Supplementary figures**

**Figure S1: Primer sets used for the qRT-PCR and RT-PCR analysis.**

(A) All primer sequences are listed in supplemental file 1. (B) For the qRT-PCR analysis presented in Figure 2D, primers (red) NC/C-F & NC-R, NC/C-F & C-R and NC-F& NC/C-R were used for *srpNC^2^*, *srpC* and *srpNC^1^* respectively. (C) The primer (blue) combination *srp-F1* & *srp-R1* was used for the RT-PCR analysis presented in Figure 2A and for *srp^ΔsrpC^* mutant analysis (shown in this section). (D) *srp-F2* & *srp-R2* primer combination was used for the RT-PCR analysis of the *srp^ΔsrpNC^* mutant (shown in this section). Note that there is only a 45 nucleotides (nt) difference in size between the two mRNA isoforms.

**Figure S2: SrpC, but not SrpNC, is required for *Drosophila* development.**

Table showing the percentage of embryos surviving to first, third instar larval stages and adulthood. The analyzed genotypes are indicated. *srp^6G^ is* an amorphic allele that corresponds to a mutation inducing a premature stop codon in the protein region encoded by the *srp* second exon, *srp^3^* is an amorphic allele with a mutation in the C-ZnF domain that inhibits both SrpC and SrpNC interaction with DNA and *srp^AS^* is a P-element induced mutant, which specifically affects embryonic blood cell development (Rehorn et al., 1996). In each condition, the *srpC* and *srpNC* genomic copy number is mentioned.

**Figure S3: Specific downregulation of *srpC* by RNAi.**

qRT-PCR analysis of *srpNC* (A) and *srpC* (B) products in embryos containing the ubiquitous *Tubulin-Gal4* driver (*Tub-Gal4)* alone or in combination with *srpC* RNAi (*Tub-Gal:;UAS-srpC-*RNAi). Induction of *srpC* RNAi reduces specifically (A) and efficiently (B) the *srpC* isoform level. qRT-PCR values are normalized to rp49 mRNA expression. Error bars represent standard deviation for three independent biological replicates. P-values are calculated from two-tailed t-test. (C) Kaplan-Meier survival curves of wild-type (grey), *srp^ΔsrpC^* (orange, loss-of-function of *srpC* isoform mutant), *Tub-Gal* (black, control) and *Tub-Gal; UAS-srpC-*RNAi (pink, RNAi downregulation of *srpC* mRNA) embryos. RNAi downregulation of *srpC* mRNA (pink) induces a drop in viability similar to that observed for *srp^ΔsrpC^* mutants (orange). P-values represent results of a Gehan-Breslon-Wilcoxon test comparing the wild-type with each of the different conditions, indicated by their specific colors (n=60 for each condition). (D-S) Whole mount *in situ* hybridization of mRNAs expressed in mature fat body cells, *Glutactin* (*Glt*, D-K) and plasmatocytes, *Peroxidasin* (*Pxn*, L-S) in wild-type, *srp^ΔsrpC^*, *Tub-Gal* and *Tub-Gal: UAS-srpC-*RNAi embryos of stage 16 (genotypes are indicated at the top of each column). RNAi downregulation of *srpC* mRNA (*Tub-Gal: UAS-srpC-*RNAi) has a similar impact on fat body development (D-K ; outlined by black arrowheads; red arrows indicate fat body defects) and plasmatocyte specific *Pxn* expression (L-S) as observed in *srp^ΔsrpC^* mutant embryos (E, I for fat body; M and Q for *Pxn* expression) compared with wild-type (D,H, and L, P) and *Tub-Gal* control (F, J and N, R) embryos.

**Figure S4: SrpC function is required during fat body formation, embryonic plasmatocytes and crystal cell development.**

*In situ* hybridization for *Glutactin* (*Glt*) RNA at stage 16 (A-P) and *Peroxidasin* (*Pxn,* Q-X) mRNA expression detected by *in situ* hybridization at stage 13 (Q-T) and 16 (U-X). For each genotype, the same embryo is shown in dorsal (A-D, I-L) and lateral (E-H, M-P) views. Fat body tissues expressing *Glt* (between arrowheads) are completely absent in homozygote null allele *srp^6G^* mutant embryos (D, H), are barely detected in hemizygous *srp^ΔsrpC^/srp^6G^* embryos with one copy of *srpNC* (C, G), are strongly reduced in homozygous *srp^ΔsrpC^* with two copies of *srpNC* (B, F, J, N) compared to *srp^ΔsrpC^/ srp^AS^* (K, O), *srp^AS^* homozygous (L, P) and wild-type (A, E, I, M) embryos. *Glt* expression is also detected in plasmatocytes (arrows in I-K, M-O) in wild-type, *srp^ΔsrpC^, srp^ΔsrpC^/srp^AS^* embryos, but not in *srp^AS^* embryos, which produce no embryonic hemocytes (L, P, T, X). In contrast, *pxn* expression is detected only in wild-type embryos (arrows in Q, U), while *srp^ΔsrpC^, srp^ΔsrpC^/srp^AS^* and *srp^AS^* embryos lack *Pxn* expression (R-T, V-X). *Prophenoloxidase2* (PPO2) mRNA expression in mature crystal cells (arrow in Y and Z) in wild-type (Y), *srp^ΔsrpC^*/ *srp^AS^* (Z) and *srp^AS^* embryos (A’). The number of *PPO2* expressing cells is strongly reduced in *srp^ΔsrpC^/ srp^AS^* embryos (Z) and completely absent in *srp^AS^* embryos (A’).

**Figure S5: SrpNC/Ush interaction, but not SrpC, is essential for female fly fertility.**

Tables showing number of adult offspring obtained from female flies of indicated genotypes. *srp^ΔsrpNC^* and *srp^V735G^* flies and flies having *srpNC* or *ush* downregulated by RNAi in the ovarian follicle cells using the *Traffic Jam* (*Tj*) driver (*Tj-Gal4*) produce no viable progeny. RNAi downregulation of *srpC* in female flies using *Tj-Gal4* does not affect their fertility.
